# Re-evaluating the phylogenetic relationships of zosterophylls with a comprehensively sampled dataset and a combination of traditional and new alternative methods

**DOI:** 10.1093/aob/mcaf146

**Published:** 2025-07-10

**Authors:** Pénélope Claisse, Borja Cascales-Miñana, Eliott Capel, Alexandru M F Tomescu

**Affiliations:** University of Lille, CNRS, UMR 8198, Evo-Eco-Paleo, Lille F-59000, France; CNRS, University of Lille, UMR 8198, Evo-Eco-Paleo, Lille F-59000, France; University of Lille, CNRS, UMR 8198, Evo-Eco-Paleo, Lille F-59000, France; Evolution and Diversity Dynamics Lab, University of Liège, Liège 4000, Belgium; Department of Biological Sciences, California State Polytechnic University Humboldt, Arcata, CA 95521, USA

**Keywords:** Devonian, Lycopsida, Lycophytina, fossil, phylogeny, zosterophyll, evolution

## Abstract

**Background and Aims:**

The known diversity of zosterophylls, the dominant tracheophytes at the beginning of the Devonian, has expanded significantly due to discoveries that intervened since the latest large phylogenetic treatment targeting the group, almost 30 years ago. Past phylogenetic treatments reached incomplete resolution or conflicting results on relationships among zosterophylls and between zosterophylls and lycopsids (which are traditionally thought to have evolved from among zosterophylls). This state of knowledge emphasizes the need for re-evaluation of zosterophyll relationships. Here, we address the phylogenetic relationships of zosterophylls based on the most extensive taxonomic sampling of the group and explore the evolutionary implications of the results.

**Methods:**

We assembled and analysed a data matrix of 44 taxa (38 of them zosterophylls) and 42 morphological characters using parsimony and Bayesian approaches. We evaluated the resulting topologies using a new approach aimed at reconciling phylogenetic conflict and identifying shared patterns of relationships, alongside traditional stratigraphic fit tests and ancestral character state reconstruction.

**Key Results:**

Within the Lycophytina clade, zosterophylls form a grade paraphyletic to the lycopsids. A Sawdoniaceae clade of zosterophylls is sister to the lycopsids; the Sawdoniaceae–lycopsids clade is sister to a clade that includes the barinophytes.

**Conclusions:**

A major radiation before and during the late Silurian gave rise to the main zosterophyll lineages (including lycopsids) and led to diversification within each of these lineages. Cladogenesis continued through the Lochkovian and relented in the Pragian, with virtually no diversification afterwards. Lycophytes (Lycophytina) are ancestrally defined by bivalvate sporangia opening along their distal margin, attached laterally on axes. Lycophytina comprises two clades: one characterized by irregularly distributed sporangia and the other (including the majority of zosterophylls, along with lycopsids and barinophytes) characterized by reniform sporangia arranged in rows, 3-D branching architectures and K-/H-branching in the rhizomatous portions.

## INTRODUCTION

The zosterophylls are an emblematic group of early land plants with worldwide distribution, and the major component of the Devonian eophytic flora (Cleal and Cascales-Miñana, 2014, 2021; Capel *et al*., 2021). Compared with other major groups of early land plants, zosterophylls were particularly abundant during the Early Devonian, from Europe (e.g. Gerrienne, 1988, 1996*a*, *b*) to Asia (e.g. Hao and Xue, 2013), Australia (e.g. McSweeney *et al*., 2020) and North America (e.g. Hueber, 1971*a*; Gensel, 1982; Gensel and Berry, 2016). The group was designated initially as subdivision Zosterophyllophytina of division Tracheophyta by Banks (1968), in a seminal paper aimed at clarifying the poorly understood relationships among early vascular plants, of which few were known at the time. Morphologically, they were initially defined by leafless axes with laterally attached sporangia and exarch xylem (Banks, 1968, 1975).

Since the publication of Banks’ oft-cited classification, more than 40 additional genera have been assigned to the group, expanding considerably its morphological breadth, with implications for how the group is defined. This is because, alongside ‘typical’ forms (i.e. conforming to Banks’ definition of the group), a number of taxa present more unusual morphologies, such as *Konioria* (Zdebska, 1982), with individual sporangia located at points of axis dichotomy, and *Xitunia* (Xue, 2009), a plant with relatively large spike-like extensions on the sporangia. As a result of this dramatic expansion of known zosterophyll diversity, the group has become difficult to define in a taxonomic context.

New fossil discoveries in the past two decades, many of them from South China (Xu *et al*., 2011; Zhu *et al*., 2011; Hao and Xue, 2013; Edwards *et al*., 2016; Wang *et al*., 2018, 2020; Edwards and Li, 2018*b*), prompted studies that assessed the patterns of diversity in the group (Cascales-Miñana and Meyer-Berthaud, 2015; Edwards and Li, 2018*a*). However, despite the large amount of recent data (additional taxa, additional information on previously recognized taxa), an updated zosterophyll phylogeny including all or most of the new taxa is still lacking. In fact, the first study to address relationships among zosterophylls in a phylogenetic context, conducted by Gensel (1992), included a limited number of taxa and characters. In her study, Gensel defined two groups among zosterophylls: the ‘Sawdoniales’, consisting of *Sawdonia* (Hueber, 1971*b*), *Crenaticaulis* (Banks and Davis, 1969), *Gosslingia* (Edwards, 1970), *Tarella* (Edwards and Kenrick, 1986), *Konioria* and *Thrinkophyton* (Kenrick and Edwards, 1988*a*), and the ‘pre-lycopsids’.

Subsequently, in an extensive study that remains a key reference almost 30 years later, Kenrick and Crane (1997) laid the foundations of the currently accepted understanding of early tracheophyte phylogeny. Kenrick and Crane's results supported a monophyletic subdivision Euphyllophytina sister to a clade (subdivision Lycophytina) that included class Zosterophyllopsida and class Lycopsida. They defined Zosterophyllopsida based on axes with circinate (spirally coiled) apices and laterally attached sporangia arranged in two rows. Kenrick and Crane (1997) further noted groups with distinguishing features within Zosterophyllopsida, including the enigmatic Barinophytaceae, recognized for their heterospory and ‘sporangiferous appendages’, and the Sawdoniaceae, characterized by the presence of emergences on the axes. However, despite providing a much-needed benchmark, Kenrick and Crane's results also revealed issues in phylogenetic resolution. For instance, some zosterophylls, such as *Hicklingia* (Kidston and Lang, 1924), *Discalis* (Hao, 1989*b*), *Gumuia* (Hao, 1989*a*) and some species of *Zosterophyllum* (Penhallow, 1892), were recovered in a polytomy with the Lycopsida and Zosterophyllopsida clades, raising questions about the monophyly of zosterophylls. Moreover, the sampling of Kenrick and Crane's study was constrained by the diversity of taxa known at that time, most of which came from Europe and North America.

This work was followed by other phylogenetic analyses (Hao and Xue, 2013; Nibbelink and Tomescu, 2022), but none of these included all (or even the majority) of the zosterophyll diversity known at the respective time. For example, the analysis by Hao and Xue (2013) focused mainly on the taxa discovered in the Posongchong Formation of China. These authors found support for a pattern of relationships different from those of previous studies, with Zosterophyllopsida a monophyletic group distinct from Lycopsida. Based on Hao and Xue's results, Zosterophyllopsida is defined by an elliptical or strap-shaped primary xylem strand with exarch maturation, an absence of perforations in the thickening bars of the tracheids and a rowed arrangement of lateral sporangia. In Hao and Xue’s (2013) analyses, the two clades, Zosterophyllopsida and Lycopsida, are part of a grade paraphyletic to Euphyllophytina. The latest analysis of zosterophylls (Nibbelink and Tomescu, 2022) explored the influence of anatomical characters on the resolution of phylogenetic relationships. As a result of their approach, taxon sampling in that study was constrained by the availability of anatomical data. Nibbelink and Tomescu's analysis found consistent support for two clades that included the relatively small number of zosterophylls sampled, with Lycopsida nested in a clade that also included *Ventarura* (Powell *et al*., 1999).

In summary, previous analyses of zosterophyll relationships sampled different subsets of taxa and support incongruent patterns of relationships. In the absence of a consistent phylogenetic framework, there is confusion on the taxonomy of the group, with the result that each zosterophyll study has defined the group as needed for its own purposes. One such example is the study of plant diversity over time by Cascales-Miñana and Meyer-Berthaud (2014), in which the authors used their own definitions of Zosterophyllopsida *sensu stricto*, which included only zosterophylls and closely allied taxa, and Zosterophyllopsida *sensu lato*, which also included the Barinophytaceae. Thus, providing a solid phylogenetic framework is important for studies of the group. Furthermore, numerous zosterophylls have autapomorphic characters that confuse understanding of their relationships. This, coupled with the significant slice of zosterophyll diversity uncovered by the more recent discoveries, has created a need for redefining the group, and reassessing relationships both within the group and with other groups of tracheophytes. Here, we aim to (1) test whether zosterophylls are monophyletic, (2) elucidate the evolutionary relationships of lycophytes and zosterophylls, and (3) shed light on the tempo and mode of lycophyte and zosterophyll evolution.

## MATERIALS AND METHODS

### Taxon sampling

We prepared a phylogenetic matrix of morphological and anatomical characters including 44 Silurian and Devonian genera (Supplementary Data Methods S1). To polarize the analysis, outgroups comprise coeval non-zosterophyll taxa: the protracheophyte *Aglaophyton* (Edwards, 1986) and the paratracheophyte *Rhynia* (Kidston and Lang, 1920*a*); trees were rooted in *Aglaophyton* in all the analyses. Two lycopsids, *Asteroxylon* (Kidston and Lang, 1920*b*) and *Drepanophycus* (Göppert, 1852), and two euphyllophytes, *Psilophyton* (Dawson, 1859) and *Polythecophyton* (Hao *et al*., 2001), are also included for testing relationships between zosterophylls and other groups.

Zosterophylls and related taxa were sampled extensively. For instance, our dataset includes 15 taxa that were not previously integrated in any phylogenetic analysis: *Baoyinia* (Edwards and Li, 2018b), *Bathurstia* (Hueber, 1971a), *Craswallia* (Morris and Edwards, 2014), *Demersatheca* (Li and Edwards, 1996), *Euthursophyton* (Mustafa, 1978), *Forania* (Jensen and Gensel, 2013), *Gosferia* (Gerrienne, 1991, 1999), *Guangnania* (Wang and Hao, 2002), *Gutzeitia* (Snigirevsky *et al*., 2007), *Macivera* (Kotyk *et al*., 2002), *Odonax* (Gerrienne, 1996b), *Omniastrobus* (Bonacorsi *et al*., 2021), *Ornicephalum* (Edwards and Li, 2018a), *Sichuania* (Edwards and Li, 2018b) and *Xitunia* (Xue, 2009) (see Supplementary Data Methods S1 for further information on taxa). These taxa are included alongside the 22 other genera of zosterophylls, representing the most extensive sampling of the group for a single analysis, to date. We did not include in the analysis genera viewed as putative zosterophylls but considered *incertae sedis*: *Bracteophyton* (Wang and Hao, 2004), *Danziella* (Edwards, 2006), *Dibracophyton* (Hao *et al*., 2012), *Faironella* (Gerrienne, 1996*a*), *Jugumella* (Senkevich, 1978), *Huia* (Geng, 1985), *Hsua* (Li, 1982), *Krithodeophyton* (Edwards, 1968), *Nothia* (El-Saadawy and Lacey, 1979) and *Renalia* (Gensel, 1976). We also excluded genera known from single, poorly preserved specimens, such as *Parazosterophyllum* and *Gippslandites* (McSweeney *et al*., 2020); see Supplementary Data Methods S2 for further information on taxa not included in the analysis. While the majority of the genera included in this study are monotypic, *Guangnania* has two species (Wang and Hao, 2002; Edwards *et al*., 2016 ) and *Sawdonia* has at least three currently accepted species (Gerrienne, 1996*a* ; Gensel and Berry, 2016; Berry and Gensel, 2019); in their cases, character scoring took into account all the species. In contrast, *Zosterophyllum* and *Psilophyton* each include multiple species. However, because these genera may be paraphyletic or polyphyletic (Kenrick and Crane, 1997; Hao and Xue, 2013; Nibbelink and Tomescu, 2022) and resolving relationships among their diverse species falls beyond the scope of our study, which focuses on genus-level relationships, we only included for each of the two genera one of the most completely characterized species: *Zosterophyllum myretonianum* (Penhallow, 1892 ) and *Psilophyton crenulatum* (Doran, 1980).

### Character construction and scoring

**Fig. 1.**
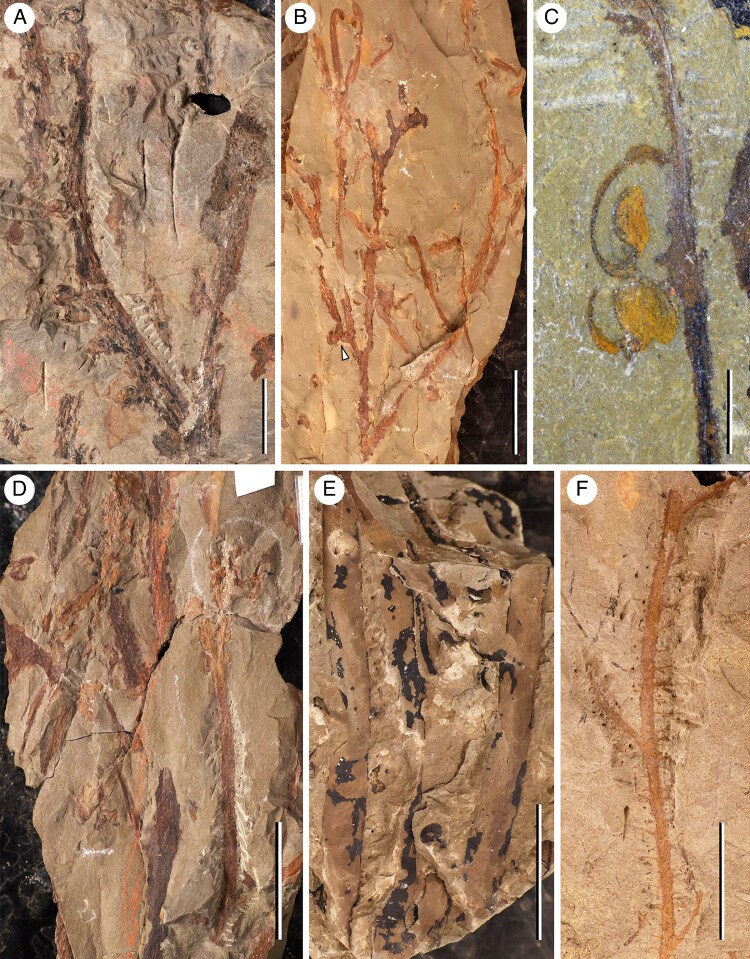
Zosterophyll morphology. (A) *Sawdonia deblondii* axis with dichotomy (bottom) bearing lateral sporangia (left of axis). Collections of the University of Liège, Belgium, ULg 13404E. Scale bar = 2 cm. (B) *Faironella valentula* (not included in this study). Multiple dichotomizing axes showing small surface emergences and branching pattern where branches form a curve apically close to their base and become more or less parallel to subtending axes; note K-branching (arrowhead) and circinate axis tips (at top left). ULg 13367. Scale bar = 2 cm. (C) *Gosferia curvata* axis bearing two sporangia on long coiled stalks. ULg 12332. Scale bar = 5 mm. (D) *Odonax borealis* axes. Note closely spaced sporangia on axis at right (top right, in circle traced in white). ULg 13361. Scale bar = 1 cm. (E) *Oricilla bilinearis* smooth axes; unnumbered specimen in the University of Liège collections. Scale bar = 2 cm. (F) *Anisophyton* cf. *gothanii* axis showing dichotomies and fine long surface emergences. ULg 13393. Scale bar = 2 cm.

**Fig. 2.**
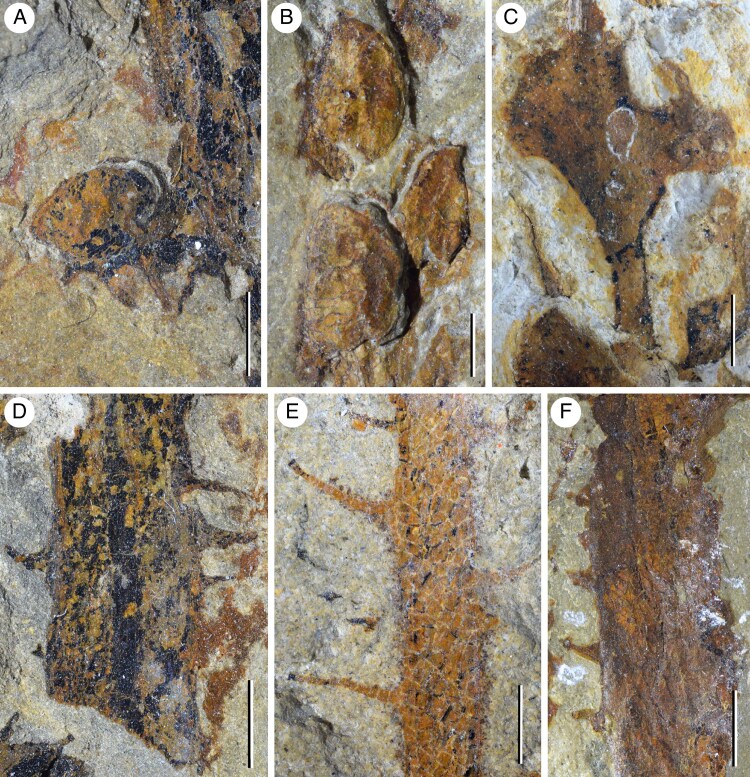
Zosterophyll morphology. (A) *Sawdonia deblondii* sporangium with spinescent emergences on bottom side (detail of Fig. 1A). Collections of the University of Liège, Belgium, ULg 13404E. Scale bar = 3 mm. (B) *Odonax borealis* closely spaced sporangia (detail of Fig. 1D). ULg 13361. Scale bar = 2 mm. (C) *Adoketophyton subverticillatum* appendage of a strobilus (detail of Fig. 3C). Collections of the Institute of Botany, Academia Sinica, Beijing, China, CBYn9002015. Scale bar = 2 mm. (D) *Sawdonia deblondii* surface emergences. ULg 13477. Scale bar = 5 mm. (E) *Anisophyton* cf. *gothanii* surface emergences (detail of Fig. 1F). ULg 13393. Scale bar = 5 mm. (F) *Odonax borealis* surface emergences (detail of Fig. 1D). ULg 13361. Scale bar = 4 mm.

**Fig. 3.**
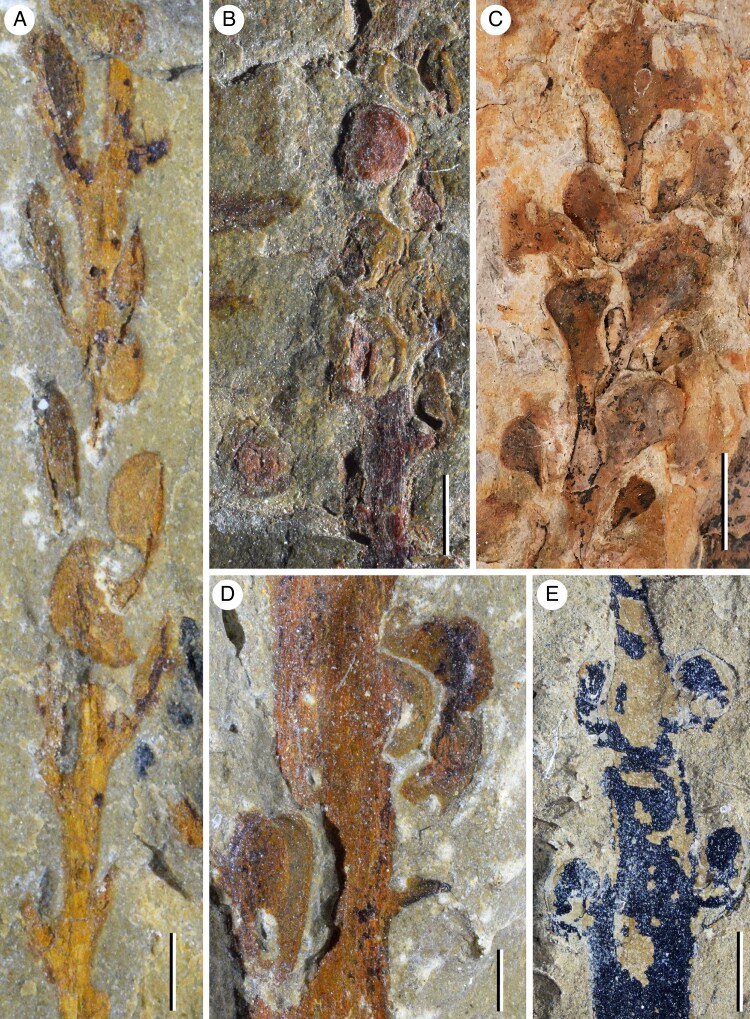
Zosterophyll morphology. (A) *Zosterophyllum deciduum* fertile axis with sporangia. Collections of the University of Liège, Belgium, ULg 13155. Scale bar = 4 mm. (B) *Zosterophyllum* fertile axis with closely spaced sporangia (strobilus). ULg 1485. Scale bar = 2 mm. (C) *Adoketophyton subverticillatum* axis with closely spaced appendages of a strobilus. Collections of the Institute of Botany, Academia Sinica, Beijing, China, CBYn9002015. Scale bar = 1 cm. (D) *Faironella valentula* (not included in this study) sporangia and surface emergence. ULg 13367. Scale bar = 5 mm. (E) *Oricilla bilinearis* sporangia. Unnumbered specimen photographed while on loan to the University of Liège collections. Scale bar = 2 mm.

Most characters are based on those previously defined by Kenrick and Crane (1997), Hao and Xue (2013) and Nibbelink and Tomescu (2022) , with some modifications, but the matrix also includes ten new characters. The taxa were scored for the 42 characters (Supplementary Data Sheets S1 and S2), which codify discrete morphological and anatomical traits (Supplementary Data Methods S3; Figs 1–3 illustrate zosterophyll morphological features), using information published in the literature. The characters were treated as unordered. The resulting matrix of 44 taxa and 42 characters (http://morphobank.org/permalink/?P4938; Supplementary Data Sheets S1 and S2) has 20 % missing data.

### Tree search methods

All previous cladistic analyses of zosterophyll relationships have employed parsimony as the optimality criterion (Gensel, 1992; Kenrick and Crane, 1997; Hao and Xue, 2013; Nibbelink and Tomescu, 2022). However, model-based approaches to phylogenetic questions have also been applied in palaeontology (Ronquist *et al*., 2012; Wright, 2019). Here, we are using both parsimony and a Bayesian approach in addressing zosterophyll relationships.

Parsimony analyses were conducted in TNT 1.5 (Goloboff and Catalano, 2016) using a traditional search (command *mult*). Bayesian analyses were conducted using MrBayes 3.2.7a (Huelsenbeck and Ronquist, 2001). The analyses were run under a Markov (Mkv) model (Lewis, 2001), with 1 000 000 generations, 50 % burn-in, two parallel runs and four chains, in order to ensure broad exploration of the tree space. Convergence was verified by the 0.012737 value for the deviation of split frequencies in MrBayes and effective sample size values of 613 and 678 (estimated using Tracer; http://beast.community/tracer.html). Since the consensus trees obtained in the Bayesian analyses had very low phylogenetic resolution (Supplementary Data Fig. S1), we computed the all-compat consensus (ACC) tree (generated using the *contype* = *allcompat* option in the *sumt* command), which shows only the fully compatible clades across the posterior distribution, thus representing the most strongly supported phylogenetic relationships across all sampled trees in the Bayesian analysis. We used this ACC tree as a counterpart to the results of the parsimony analysis for the purpose of introducing new ways of assessing phylogenetic relationships in contexts where different tree-search methods yield topologies with low degrees of congruence.

### Taxon exclusion experiments

High taxon-to-character ratios are more likely to decrease phylogenetic resolution (e.g. Scotland *et al*., 2003). Additionally, some of the zosterophyll taxa are known only from fragmentary material and have high proportions of missing data, another factor known to lower phylogenetic resolution (e.g. Wiens, 2003). For these reasons and in order to improve the level of resolution obtained by the parsimony analysis with full taxon sampling (FTS), we conducted several analyses under parsimony with different subsets of taxa excluded. The choice of taxa for the exclusion experiments was driven by their amounts of missing data, looking for the (consensus) trees with the highest amount of resolution for the lowest number of excluded taxa; taxa excluded in those analyses were those with the highest proportions of missing data: *Euthursophyton* (Mustafa, 1978) (64.3 % missing data), *Craswallia* (40.5 %), *Xitunia* (40.5 %), *Gutzeitia* (38.1 %), *Trichopherophyton* (Lyon and Edwards, 1991) (38.1 %), *Gosferia* (35.7 %) and *Baoyinia* (35.7 %), in different numbers and combinations. Taxon exclusion in most of these analyses improved phylogenetic resolution compared with the FTS analysis. The best resolution – a fully resolved most parsimonious (MP) tree – was obtained when only two taxa (*Gutzeitia* and *Gosferia*) were excluded (GGE tree). Our approach to identifying taxa that lower the amount of phylogenetic resolution (referred to in the literature as wildcard taxa and rogue taxa) did not follow methods developed specifically for this (e.g. Smith, 2022 ) and instead we inspected strict consensus trees obtained under different numbers and combinations of excluded taxa, because (1) we were not directly interested in identifying rogue taxa but in seeing how, specifically, exclusion of different combinations of taxa might alter the supported relationships among remaining taxa; and (2) because in applying this approach we serendipitously stumbled over a combination of only two taxa which, when excluded, provided maximum phylogenetic resolution.

### Phylogenetic congruence, taxon stability and cohorts of phylogenetic divergence

Phylogenetic congruence, taxon stability and the cohorts of phylogenetic divergence were assessed by comparing the phylogenetic relationships supported by the parsimony and the Bayesian analyses performed in the GGE taxon sampling regime, as reflected in (1) the single MP tree obtained in the parsimony analysis and (2) the ACC tree obtained in the corresponding Bayesian analysis.

To the best of our knowledge, the only method developed for quantifying *phylogenetic congruence*, i.e. the overall similarity between two phylogenetic trees that include the same selection of taxa, involves the set of metrics derived by comparing quartet types (Estabrook *et al*., 1985; Estabrook, 1992). Because that method is computationally intensive and we were looking for a measure of congruence not as a goal in and of itself, but only in order to provide some context to the analyses of taxon stability and cohorts of phylogenetic divergence, we chose to quantify phylogenetic congruence in a simpler way, as the proportion of nodes that are supported in both trees out of the total nodes on each of the two fully resolved trees.

Because the two trees inferred using parsimony and the Bayesian approach are not entirely congruent, so different taxa occupy different positions in the two trees, we needed a way to quantify these differences, which led to our development of a new metric of taxon stability. Here, taxon stability refers to a measure of positional congruence of individual taxa between two phylogenies computed with inclusion of those taxa and containing the same taxa, in other words the amount that their placements differ between the two phylogenies. This is not the only meaning that was given to this or related concepts. For instance, Klopfstein and Spasojevic (2019) developed RoguePlots to quantify (and illustrate) placement uncertainty, or the confidence in the *a posteriori* placement of rogue taxa on phylogenetic backbones computed without inclusion of those taxa. In another example, Smith (2022) proposed a measure of taxon instability that quantifies the stability of a taxon as a function of its position relative to each of the other taxa and compared among all the trees in the phylogenetic tree population obtained using the same method of analysis under different taxon exclusion regimes. This is different from our concept (and metric, which we introduce below) of taxon stability, which compares the placement of a taxon between two phylogenetic trees that contain the same number of taxa, and in terms of its placement relative exclusively to the hierarchy of branching of those trees and not relative to other taxa. More similar to our approach to taxon stability, Estabrook (1992) calculated the positional congruence of individual taxa between two different phylogenetic trees that include the same taxa. However, Estabrook uses the stability of individual taxa as a measure of incongruence between those two trees, and not as a means to compare stability among taxa. Furthermore, like Smith’s (2022) taxon instability metric and unlike the metric we introduce below, Estabrook's metric for positional congruence is based on quartet types and is, thus, dependent on the position of the taxon in question relative to all the other taxa in the two trees.

Our approach to quantifying taxon stability begins with assigning each taxon in each of the two trees a number corresponding to the rank of the node from which it diverged in that tree (*R*). *Node ranks* were numbered from the base towards the crown of the tree, in our specific case starting with the basal node of Lycophytina (the sister clade to the euphyllophytes), which was assigned rank 1; from there, node ranks were numbered up the tree, with the nodes immediately succeeding node of rank *n* in a clade being assigned the rank *n* + *1* on each of the two lineages that diverge from node *n*, so the higher a node is in a tree, the higher its rank. To facilitate direct comparisons between the positions of a taxon on the two trees, we normalized the number of node ranks (*R*^+^) in the tree with a higher number of ranks (*h* = 15 node ranks in our maximum parsimony tree) to match the number of node ranks (*R*^−^) in the tree with fewer node ranks (*f* = 13 ranks in our Bayesian ACC tree): norm*R*^+^ = (*R*^+^ × *f*)/*h*.

The new metric of *taxon stability* (TS) was calculated for each taxon as the positive value of the difference between the ranks of the nodes at which that taxon diverges in the two trees: TS = *R*^−^ − norm*R*^+^ (see Supplementary Data Methods S4 for an example of taxon stability calculation). Thus, the most stable taxa diverge from nodes of the same or closely similar ranks in both trees (TS equal or close to 0) and the higher the TS value of a taxon the less stable its phylogenetic position. For simplicity, pairs of sister taxa that are supported in both analyses are each treated as a single lineage in the analyses of taxon stability and of the cohorts of phylogenetic divergence.

In summary, like other previously developed metrics (Estabrook, 1992; Klopfstein and Spasojevic, 2019; Smith, 2022), our measure of taxon stability quantifies the positional congruence of a taxon between phylogenetic trees where it occupies different positions. This allows the identification of taxa whose missing data or combinations of plesiomorphic character states lower phylogenetic resolution or increase homoplasy, while also providing improved understanding of the more stable or better supported regions of phylogenetic trees, from a qualitative standpoint (e.g. those regions where more stable taxa are concentrated). On the other hand, our metric is original in adressing several aspects that are not combined in any previously developed metric of taxon stability: (1) it is based on comparison of trees that include the same taxa; (2) based on comparison of trees computed including the taxa in question; and (3) it is computed based on taxon position as quantified by the rank of the node of divergence and independent of the positions of the other taxa in the trees.

The ranks of the nodes at which each taxon diverges were also used to identify *cohorts of phylogenetic divergence*, another new concept introduced here. Because phylogenetic trees depict sequences of cladogenetic events against a relative time scale, the relative positions of nodes on a tree reflect sequences of divergence. As a result, the rank of the node at which a taxon diverges provides a relative measure of how derived the taxon is within that specific phylogeny. The cohorts of phylogenetic divergence group taxa diverging at nodes of similar ranks and, therefore, can be construed to represent roughly equivalent levels of morphological evolution, irrespective of how closely they are related phylogenetically. This concept thus provides a broad-brush picture of major evolutionary patterns and can be useful especially in cases where phylogenetic relationships supported by different methods of inference (for the same taxon sampling) involve some degree of incongruence. In such incongruence cases, where some taxa are placed in different positions on the different trees that are compared, each taxon has a range of node ranks at which it diverges in the different trees; the range is narrower for the more phylogenetically stable taxa and broader for less stable taxa. The cohorts are formed by considering the node rank ranges of taxa (see Supplementary Data Methods S4 for an example of cohorts of phylogenetic divergence). In our case, the cohorts were formed by separating groups of taxa visually along the most obvious breaks in the taxon divergence rank graph, placing highest emphasis on the most stable taxa (TS < 1.5) and downweighing the most unstable taxa with TS ≥ 2.

### Stratigraphic fit and ancestral character state reconstruction

Ancestral character state reconstruction was performed in Mesquite (Maddison and Maddison, 2022) on the strict consensus tree from the FTS analysis. We employed a maximum likelihood probability model to infer the ancestral character state at a node, which was the character state with >50 % probability. Ancestral state reconstruction was performed for four characters selected because of their relevance to discussions on the taxonomic circumscription and the evolution of zosterophylls and lycophytes (e.g. Kenrick and Crane, 1997; Gerrienne *et al*., 2016). The characters are circinate axis tips (character 6), sporangium shape (character 20), relative sporangium valve size (character 25) and sporangium arrangement along axes (character 33).

The stratigraphic fit analysis was conducted using the R package *strap* (Bell and Lloyd, 2015). The stratigraphic ranges and ages (Supplementary Data Methods S1 and Supplementary Data Sheet S3) were based only on the distribution of species of each genus that are not published in open nomenclature (so, for example, the occurrences of cf. *Distichophytum* sp. and *Bathurstia* sp. in the Ludlow of Canada – Kotyk *et al*. (2002) – were not considered in determining the stratigraphic ranges of those genera; similarly, occurrences of *Sawdonia* in the UK and Poland, currently treated as cf. *Sawdonia* sp. following the Gensel and Berry (2016) redefinition of the genus concept, were not considered). The absolute ages used to bracket the stratigraphic ranges of each taxon were the values listed in the International Chronostratigraphic Chart (Cohen *et al*. 2013) for the lower and upper limits of the interval covered by all considered occurrences of that taxon, not including the errors associated with geochronological age; for example, if one occurrence of a taxon was dated as Early Devonian and another as Pragian–Emsian, the stratigraphic range of the taxon was considered Early Devonian, 419.2–393.3 Ma. In cases where ages were listed as subdivisions of geological stages (early, middle, late), the duration of that stage was divided accordingly into equal intervals; for example, an early Emsian stratigraphic range for a taxon was considered to span between the age of the Pragian–Emsian boundary (407.6 Ma) and the lower third of the duration of the Emsian (to 402.8 Ma).

The workflow involved time-scaling each of the MP trees produced by the FTS parsimony analysis and computation of multiple stratigraphic fit indices, which allowed selection of the MP tree with the best stratigraphic fit. This fully resolved FTS tree was used to discuss patterns of evolutionary diversification, infer ghost lineages and estimate the timing of evolutionary events. This tree was also considered in comparisons with the results of previous phylogenetic analyses and is recommended for consideration in comparisons with the results of future analyses that require fully resolved trees and broadest taxon sampling.

## RESULTS

### Parsimony analyses

**Fig. 4.**
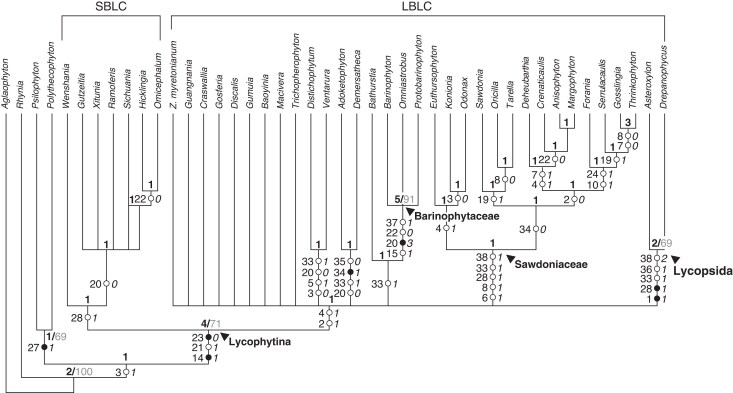
Strict consensus of six MP trees (*L* = 149; CI = 0.313; RI = 0.534) obtained in the FTS analysis. Numbers above or next to nodes are Bremer support (black, bold)/bootstrap support values >50 (grey). Black circles under nodes are hard synapomorphies; white circles are putative synapomorphies; numbers at left of dots are character numbers, numbers at right, in italics, are character states. SBLC, small basal Lycophytina clade; LBLC, large basal Lycophytina clade.

The FTS analysis recovered six MP trees (*Length (L)* = 149; Consistency Index (CI) = 0.313; Retention Index (RI) = 0.534). As is typical for analyses involving relatively low numbers of morphological characters (e.g. Sanderson, 1989; Bremer *et al*., 1999; Wilkinson, 2003), clade support values (bootstrap and Bremer; Fig. 4) are low. The strict consensus tree of the six MP trees (Fig. 4) supports the same pattern of relationships as the 50 % majority rule consensus tree. The tree separates the euphyllophytes (trimerophytes: *Psilophyton crenulatum* and *Polythecophyton*) from a Lycophytina clade that groups all the zosterophylls and the lycopsids. The clade formed by the two sister lineages (euphyllophytes and Lycophytina) is sister to *Rhynia*. The synapomorphies that support the Lycophytina clade are laterally attached sporangia (character 14) that dehisce along their distal margin (character 23) and have bilateral symmetry (character 21); the latter character presents reversals to radial symmetry in *Margophyton* and *Craswallia* and cannot be scored with certainty (missing data) in the barinophytes and *Euthursophyton*.

A basal dichotomy of Lycophytina separates two clades. The smaller of the two clades, hereafter termed the small basal Lycophytina clade (SBL clade or SBLC), includes a ((*Hicklingia*, *Ornicephalum*) *Sichuania*) clade that forms a polytomy with *Ramoferis*, *Xitunia* and *Gutzeitia*; this polytomic node is sister to *Wenshania* (Fig. 4). All the other members of Lycophytina are recovered in the second, larger basal clade, hereafter termed the large basal Lycophytina clade (LBL clade or LBLC). This clade has a large basal polytomy of lineages, some of which are clades: *Distichophytum* + *Ventarura*, *Adoketophyton* + *Demersatheca*, the Barinophytaceae + *Bathurstia*, the lycopsids (*Asteroxylon* and *Drepanophycus*), and a larger clade referred to here as the Sawdoniaceae.

The Barinophytaceae clade (*Barinophyton*, *Protobarinophyton* and *Omniastrobus*) is supported by the lenticular, vertically flattened shape of sporangia.

The Sawdoniaceae clade is supported by a number of synapomorphies, each of which either shows reversals within the clade or cannot be scored unequivocally in all the clade members: (1) circinate axis tips (character 6) and (2) emergences (character 8), (3) sporangia distributed on the highest and lower orders of branching (character 28), and (4) xylem strand with elliptical cross-sectional shape (character 38). Circinate axis tips show a reversal in *Margophyton* and the presence of emergences sees reversals in *Gosslingia*, *Thrinkophyton*, *Oricilla* and *Tarella*. The elliptical cross-sectional shape of the xylem cannot be scored (due to lack of anatomical data) in *Anisophyton*, *Odonax*, *Serrulacaulis*, *Oricilla*, *Forania* and *Tarella*, and is therefore only predicted for these taxa based on support of the character state as synapomorphic in the clade; the same is true about the distribution of sporangia (along highest and lower order branches) in *Euthursophyton* and *Serrulacaulis*. A fifth putative synapomorphy, the regular arrangement of sporangia (character 33) sees a reversal in *Forania* and cannot be scored in *Euthursophyton*; more importantly, this character is inapplicable in *Anisophyton*, *Konioria* and *Margophyton*, which indicates that its synapomorphic status in the Sawdoniaceae, supported by our analyses, is an artefact of the tree-search algorithm, which takes into account each possible state of a given character in taxa where the respective character is scored inapplicable.

Within the Sawdoniaceae, a clade that includes ((*Konioria* + *Odonax*) *Euthursophyton*) is sister to a clade in which an ((*Oricilla* + *Tarella*) *Sawdonia*) clade is sister to a clade that consists of two sister groups: one including *Gosslingia*, *Thrinkophyton*, *Serrulacaulis* and *Forania*, and the other including *Anisophyton*, *Margophyton*, *Crenaticaulis* and *Deheubarthia*.

**Fig. 5.**
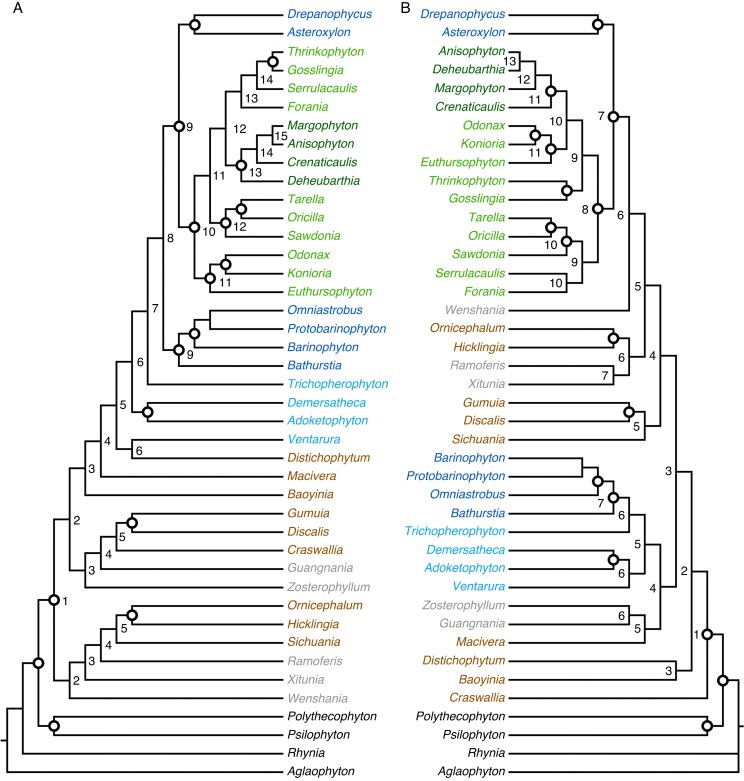
Results of the reduced taxon sampling (*Gosferia* and *Gutzeitia* excluded; GGE) analyses. (A) Single MP tree (*L* = 145; CI = 0.386; RI = 0.663) obtained in the parsimony analysis. (B) ACC tree from the Bayesian analysis. Numbers at nodes show the node ranks used in the taxon stability analysis. Circles mark nodes supported in both trees that give a measure of congruence between the results of the two methods. Colours correspond to cohorts of phylogenetic divergence (see also Fig. 7): green, highest-diverging taxa (higher-diverging subgroup in dark green, lower subgroup in light green); blue, middle cohort (higher-diverging subgroup in dark blue, lower subgroup in light blue); brown, lowest-diverging cohort; grey, lycophytes not assignable to a cohort due to high instability; black, non-lycophyte taxa.

The GGE analysis recovered the best resolution for the broadest sampling of currently recognized zosterophylls to date: a single MP tree. The GGE tree (*L* = 145; CI = 0.386; RI = 0.663; Fig. 5A) supports the same clades within Lycophytina as the FTS consensus tree (Fig. 4). The SBLC, including (((((*Hicklingia*, *Ornicephalum*) *Sichuania*) *Ramoferis*) *Xitunia*) *Wenshania*), is sister to a clade that includes all the remaining lycophytes (LBLC). In the latter, a basal split separates a clade including ((((*Discalis*, *Gumuia*) *Craswallia*) *Guangnania*) *Z. myretonianum*) from a larger clade. In this larger clade, a paraphyletic grade that consists (starting at the base) of *Baoyinia*, *Macivera*, *Distichophytum* + *Ventarura*, *Adoketophyton* + *Demersatheca* and *Trichopherophyton* leads to a clade that includes the Barinophytaceae + *Bathurstia*, the lycopsids (*Asteroxylon* and *Drepanophycus*) and the Sawdoniaceae clade, each with the same membership as in the FTS analysis. Notably, the lycopsids are sister to the Sawdoniaceae and the clade they form together is sister to the Barinophytaceae + *Bathurstia* (Fig. 5A).

**Fig. 6.**
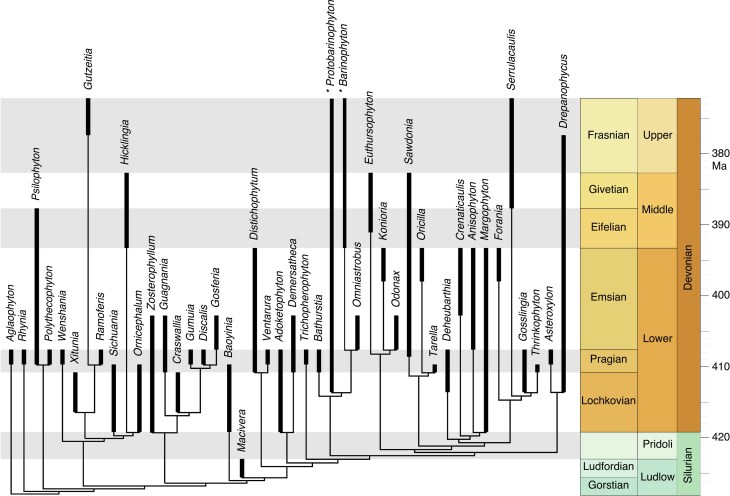
Time-scaled tree number 6 from the FTS parsimony analysis, which has the best fit to stratigraphy (see Supplementary Data Table S1 for stratigraphic fit indices and tests). The stratigraphic ranges of taxa (thicker lines) are those shown in Supplementary Data Methods; asterisks mark taxa whose ranges extend beyond the Frasnian. Chronostratigraphy based on Cohen *et al*. (2013).

The same clades and relationships supported by the GGE analysis are supported in the tree that has the best stratigraphic fit (see below) of the six MP trees recovered in the FTS analysis (BSF tree hereafter; Fig. 6). The only differences between the GGE and BSF trees concern the placement of *Gutzeitia* and *Gosferia*, which are not included in the GGE tree. In the BSF tree, *Gutzeitia* is placed in the SBLC, wherein addition of *Gutzeitia* brings *Xitunia* and *Ramoferis* into a clade (*Xitunia* sister to *Gutzeitia* + *Ramoferis*) that is sister to the (*Sichuania* (*Hicklingia*, *Ornicephalum*)) clade (Fig. 6). *Gosferia* is placed as sister to *Discalis*.

In the BSF tree, the SBLC is supported by a potential synapomorphy (sporangia distributed on both highest and lower-order branches (character 28)) that shows a reversal to sporangia borne only on highest order branches in *Gutzeitia* and cannot be scored unequivocally in *Xitunia*, *Sichuania* and *Ornicephalum*. The clade that includes *Z. myretonianum* is supported by a branching pattern in which branches curve apically close to their base to run more or less parallel with the subtending axis (character 5). However, this is only a putative synapomorphy, as missing data preclude its unequivocal scoring in *Craswallia*, *Gumuia*, *Discalis* and *Gosferia*. The large clade sister to *Macivera* has two potential synapormorphies (both of which present character state reversals or missing data in some members of the clade): roughly spherical sporangia (character 20) and sporangia irregularly arranged along axes (character 33).

### Bayesian analyses

Analyses in a Bayesian framework obtained low phylogenetic resolution. The 50 % majority rule consensus tree of the FTS analysis (Supplementary Data Fig. S1) separates the euphyllophytes from the broad Lycophytina clade and within the latter, which is highly polytomic basally, a few of the clades that are also recovered in the parsimony trees are supported: the lycopsids, the Barinophytaceae + *Bathurstia*, *Oricilla* + *Tarella* and *Adoketophyton* + *Demersatheca*. GGE taxon sampling Bayesian analyses obtain the same majority rule consensus tree as the FTS analysis.

The ACC tree computed based on the GGE Bayesian analysis also separates the euphyllophytes from the Lycophytina (Fig. 5B). This tree supports the same Sawdoniaceae clade sister to the lycopsids as the parsimony GGE analysis (Fig. 5A), but the remaining members of the Lycophytina are recovered in relationships incongruent with those supported in the parsimony GGE tree (Fig. 5).

### Phylogenetic congruence, taxon stability and cohorts of phylogenetic divergence

The relationships supported by the GGE parsimony analysis and those supported by the corresponding Bayesian analysis (as reflected in the Bayesian ACC tree) have a moderate to low level of mutual congruence: only 43.6 % (17 out of 39) of the Lycophytina nodes are supported in both trees (Fig. 5). The most notable points of congruence between the two trees are the unequivocal separation of euphyllophytes from Lycophytina (also supported in the strict consensus trees of all Bayesian analyses) and the relationship between lycopsids and Sawdoniaceae.

Taxon stability, as reflected by the ranks of the nodes at which a taxon diverges in the parsimony versus Bayesian analysis (Table 1), varies from very stable taxa (TS < 1 (*Anisophyton*, *Trichopherophyton*, *Baoyinia*, *Sawdonia*, *Oricilla* + *Tarella*, *Sichuania*, *Discalis* + *Gumuia*, *Ventarura*, *Adoketophyton* + *Demersatheca*, *Bathurstia*, Barinophytaceae and lycopsids)) to the most unstable taxa (TS ≥ 2.5 (*Guangnania*, *Gosslingia* + *Thrinkophyton*, *Craswallia*, *Z. myretonianum*, *Ramoferis*, *Wenshania* and *Xitunia*)) (Table 1). Other stable taxa (1 ≤ TS < 1.5) include *Margophyton*, *Crenaticaulis*, *Forania*, *Konioria* + *Odonax* and *Euthursophyton*, whereas less stable taxa (1.5 ≤ TS < 2.5) include *Macivera, Hicklingia* + *Ornicephalum, Deheubarthia*, *Serrulacaulis* and *Distichophytum*. The most stable taxa are *Anisophyton* (TS = 0) and *Trichopherophyton* (TS = 0.07) and the most unstable taxa (TS > 4) are *Xitunia* and *Wenshania*.

**Table 1.** Rank of node of divergence and stability of taxa included in the GGE analyses, based on the single MP tree and the ACC tree resulting from the Bayesian analysis (BayACCT); see Fig. 5 for the numbering of node ranks.

Taxon	MP	MP norm/13	BayMCCT	Taxon stability
*Adoketophyton–Demersatheca*	6	5.20	6	0.80
*Anisophyton*	15	13.00	13	0.00
*Baoyinia*	3	2.60	3	0.40
Barinophytes	9	7.80	7	0.80
*Bathurstia*	9	7.80	7	0.80
*Craswallia*	5	4.33	1	3.33
*Crenaticaulis*	14	12.13	11	1.13
*Deheubarthia*	13	11.27	13	1.73
*Discalis–Gumuia*	5	4.33	5	0.67
*Distichophytum*	6	5.20	3	2.20
*Euthursophyton*	11	9.53	11	1.47
*Forania*	13	11.27	10	1.27
*Gosslingia–Thrinkophyton*	14	12.13	9	3.13
*Guangnania*	4	3.47	6	2.53
*Hicklingia–Ornicephalum*	5	4.33	6	1.67
*Konioria–Odonax*	11	9.53	11	1.47
Lycopsids	9	7.80	7	0.80
*Macivera*	4	3.47	5	1.53
*Margophyton*	15	13.00	12	1.00
*Oricilla–Tarella*	12	10.40	10	0.40
*Ramoferis*	4	3.47	7	3.53
*Sawdonia*	12	10.40	10	0.40
*Serrulacaulis*	14	12.13	10	2.13
*Sichuania*	5	4.33	5	0.67
*Trichopherophyton*	7	6.07	6	0.07
*Ventarura*	6	5.20	6	0.80
*Wenshania*	2	1.73	6	4.27
*Xitunia*	3	2.60	7	4.40
*Zosterophyllum myretonianum*	3	2.60	6	3.40

The third column (MP norm/13) represents ranks of the nodes of divergence in the MP tree normalized to the same total number of nodes as the Bayesian tree (see text). Taxon stability is the positive value of the difference between MP norm/13 and BayACCT.

**Fig. 7.**
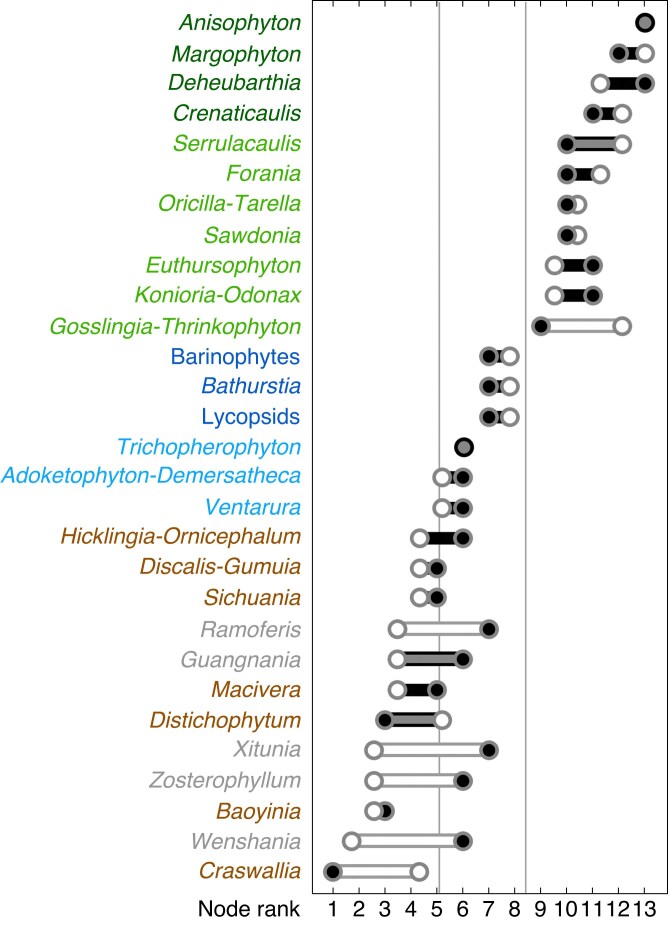
Taxon stability and cohorts of phylogenetic divergence. Taxa are organized in increasing order of the ranges of their nodes of divergence in the maximum parsimony and Bayesian GGE analyses (see Fig. 5 for node rank numbering); node ranks on the *x*-axis. Ranges of the nodes of divergence are defined by the node of divergence in the parsimony analysis (white circles) and the node of divergence in the Bayesian ACC tree (black circles) (see Table 1); grey circles indicate the same node rank in both the parsimony and the Bayesian tree. Black range bars (and grey circles) indicate taxa with high phylogenetic stability; grey bars denote less stable taxa; white bars indicate unstable taxa. The two vertical lines separate the three cohorts of phylogenetic divergence also indicated by the colour of the taxon names (see also Fig. 5): green, highest-diverging taxa (higher-diverging subgroup in dark green, lower subgroup in light green); blue, middle cohort (higher-diverging subgroup in dark blue, lower subgroup in light blue); brown, lowest-diverging cohort; grey, lycophytes not assignable to a cohort due to high instability.

Three cohorts of phylogenetic divergence were easily separated using the taxon divergence rank graph (Fig. 7). The highest cohort, comprising the most derived taxa, includes *Anisophyton*, *Margophyton*, *Crenaticaulis* and *Deheubarthia* as the top diverging taxa, as well as *Serrulacaulis*, *Forania*, *Oricilla* + *Tarella*, *Sawdonia*, *Euthursophyton* and *Konioria* + *Odonax*; *Gosslingia* + *Thrinkophyton*, despite their significant phylogenetic instability, are also undeniably part of the most derived cohort (Fig. 7). At the opposite end of the sequence of phylogenetic divergence, the lowest cohort includes many of the most phylogenetically unstable taxa; *Discalis* + *Gumuia*, *Sichuania*, *Macivera*, *Distichophytum* and *Baoyinia*; *Craswallia* is also undeniably part of the lowest cohort, despite its significant phylogenetic instability. Other unstable taxa have ranges that cross into that of the median cohort of divergence but span mostly in that of the lowest cohort – *Wenshania*, *Z. myretonianum* and *Guangnania*. On the other hand, the ranges of *Xitunia* and *Ramoferis* span almost equally into the lowest and median cohorts, so they cannot be assigned to either cohort unequivocally. *Hicklingia* + *Ornicephalum*, a stable clade, also spans into both the lowest and median cohorts (Fig. 7) and is associated with the former only because of its lower position of divergence in the parsimony analysis. Finally, the median cohort comprises a lower-diverging subgroup (*Ventarura*, *Adoketophyton* + *Demersatheca* and *Trichopherophyton*) and a higher-diverging subgroup (*Bathurstia*, Barinophytaceae and lycopsids) (Fig. 7).

Both the highest and lowest cohort include taxa of widely variable stability (from very stable to very unstable; Figs 5 and 7), suggesting there is no strong correlation between the phylogenetic stability of a taxon and its cohort of phylogenetic divergence. Nevertheless, all the taxa of the middle cohort have very high stability (TS < 1) and most of the taxa in the highest cohort are stable to very stable phylogenetically (TS < 1.5). Conversely, the lowest cohort includes the highest proportion of unstable taxa. Correspondingly, the taxa of the highest cohort maintain the same phylogenetic relationships in the parsimony and Bayesian analyses (Fig. 5), and so do the lycopsids, which are among the higher-diverging group of the median cohort, whereas the less phylogenetically stable taxa of the lowest cohort are recovered in different relationships in the parsimony versus Bayesian analyses (Fig. 5).

### Ancestral character state reconstruction

**Fig. 8.**
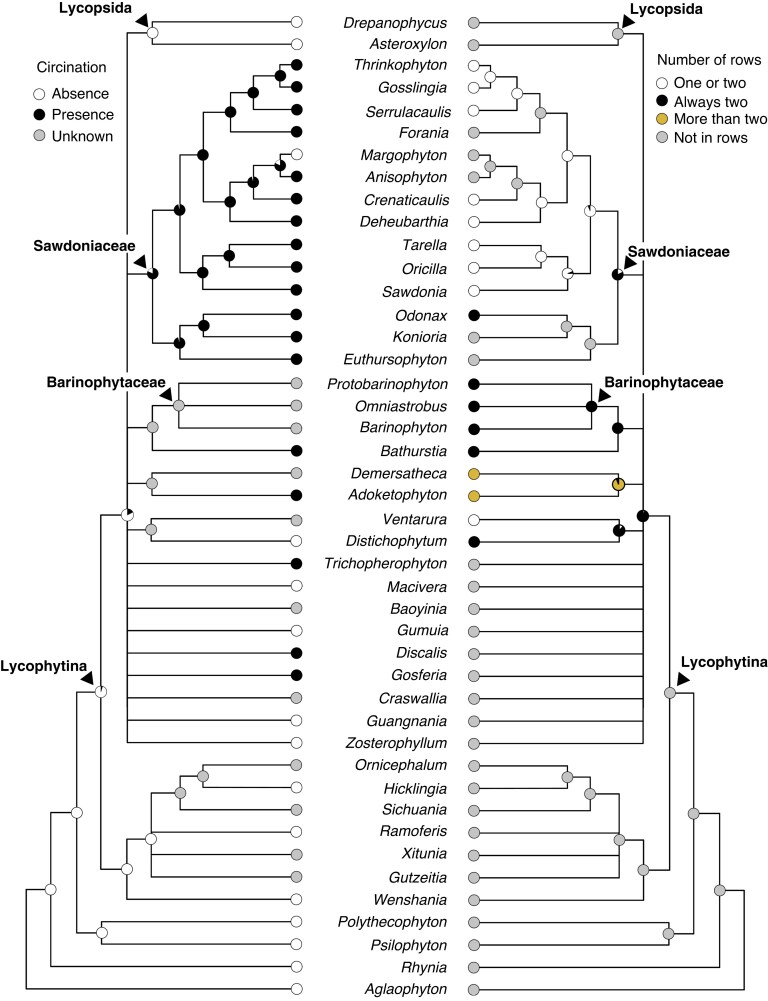
Ancestral character state reconstruction for characters 6 (presence/absence of apical circination) and 34 (sporangium arrangement in rows) based on the strict consensus topology of the FTS analysis.

**Fig. 9.**
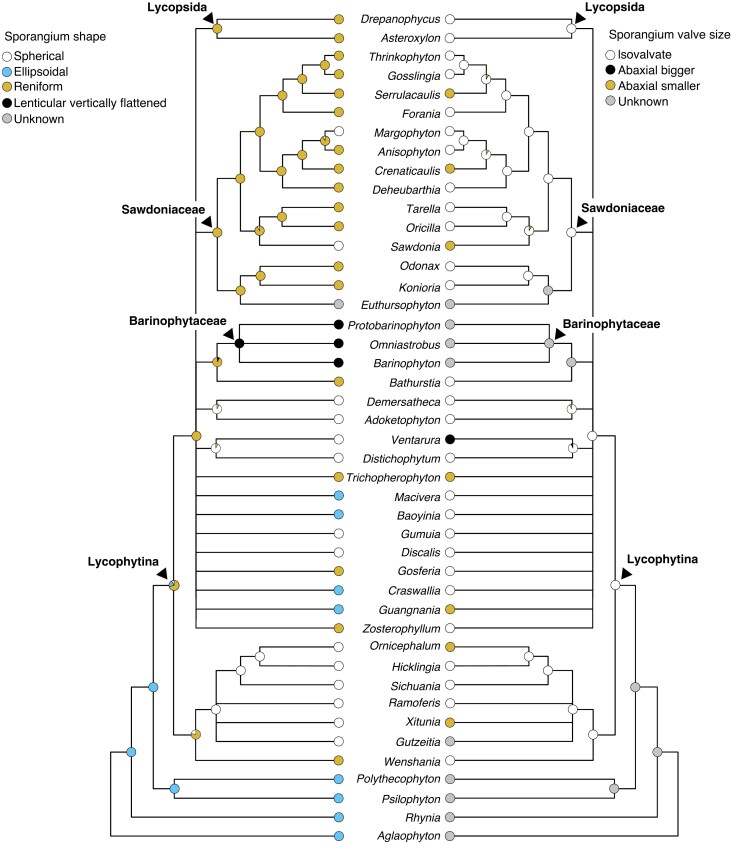
Ancestral character state reconstruction for characters 20 (sporangium shape) and 25 (sporangium valve size) based on the strict consensus topology of the FTS analysis.

Apical circination of axes is reconstructed as present in the Sawdoniaceae ancestor and absent ancestrally in Lycophytina and all its other major clades; its ancestral state is equivocal in the Barinophytaceae and in the clade that the latter form with *Bathurstia* (Table 2, Fig. 8 left). Sporangium arrangement along the axes is reconstructed as ancestrally irregular (i.e. not in rows) in Lycophytina and in the SBLC, and is also not ancestrally rowed in the lycopsids (Table 2, Fig. 8 right). The arrangement is ancestrally two-rowed in the LBLC, although it is either irregular or not necessarily two-rowed in most members of this clade. Within this clade, the Barinophytaceae and Barinophytaceae + *Bathurstia*, as well as the Sawdoniaceae are reconstructed as ancestrally possessing two-rowed sporangia, although the ancestral state is reversed in most members of the latter. Sporangium shape is reconstructed as ancestrally reniform in Lycophytina and all its major clades, except for the Barinophytaceae, in which the ancestor is reconstructed with vertically flattened lenticular sporangia like those of the three representatives of the group (Table 2, Fig. 9 left). Finally, sporangia are reconstructed as ancestrally isovalvate all across Lycophytina (Table 2, Fig. 9 right). The one exception is Barinophytaceae + *Bathurstia*, in which the ancestral state is equivocal, although this is probably an artefact of the particular shape of barinophyte sporangia, for which relative valve size could not be scored.

**Table 2.** Summary of ancestral character state reconstruction analysis.

Clade	Apical circination of axes (character 6)	Sporangium arrangement along axes (characters 33 and 34)	Sporangium shape (character 20)	Sporangium valve size (character 25)
Lycophytina	Absent	Not in rows (irregular or helical)	Reniform	Isovalvate
SBLC	Absent	Not in rows (irregular or helical)	Reniform	Isovalvate
LBLC	Absent	Always two-rowed	Reniform	Isovalvate
Barinophytaceae	Unknown	Always two-rowed	Lenticular, vertically flattened	Unknown
Sawdoniaceae	Present	Always two-rowed	Reniform	Isovalvate
Lycopsida	Absent	Not in rows (irregular or helical)	Reniform	Isovalvate

SBLC, small basal Lycophytina clade; LBLC, large basal Lycophytina clade (Fig. 4).

### Stratigraphic fit

Of the six MP trees recovered in the FTS analysis, the tree with best stratigraphic fit based on multiple measures is tree 6 (Supplementary Data Table S1). Since different groups are supported within Lycophytina, we integrated the age and stratigraphic information of the taxa (Supplementary Data Methods S1; Supplementary Data Sheet S3) with the topology of this tree to obtain a better image of their evolutionary relationships and history over time (Fig. 6).

## DISCUSSION

### The pattern of relationships among lycophytes: past and present

#### Phylogenetic resolution and relationships supported by our analyses

The low resolution of consensus trees from the full taxon sampling analyses (parsimony and Bayesian; Fig. 4, Supplementary Data Fig. S1) echoes the results of most analyses that include significant numbers of fossils. In such cases, low resolution can be caused by incomplete preservation of fossils, which allows for scoring only some of the characters (some fossils preserve only morphology, others preserve anatomy but little of the gross morphology), leading to missing data, which lowers resolution (Wilkinson, 1995; Wiens, 2003). The uneven temporal sampling of taxa, an effect of uneven stratigraphic distribution of the fossils, can be another impediment. For instance, our sampling covers densely the Pragian and Emsian intervals (Fig. 6), but there is a dearth of fossils from the preceding intervals, especially the Silurian (Capel *et al*., 2023*b*). Scarcity of older, potentially less derived taxa that would polarize characters at the base of their respective branches on phylogenetic trees could increase levels of homoplasy. Perhaps more importantly, the dearth of morphological characters – zosterophylls have simple sporophytes consisting of undifferentiated photosynthetic axes – is known to reduce phylogenetic resolution (Wiens, 2004).

All the above factors affect our matrix (1) in which the number of taxa surpasses the number of characters; (2) that has 20 % missing data; and (3) that samples unevenly the stratigraphic interval of interest. In light of these aggravating factors, the level of resolution recovered in the parsimony analysis is remarkable. Even in the FTS analysis, our dataset separates unequivocally the euphyllophytes from the lycophytes (Lycophytina), the latter into two major clades, and within one of the two clades it separates the lycopsids from Barinophytaceae + *Bathurstia* and from the Sawdoniaceae (Fig. 4).

The Bayesian consensus tree (Supplementary Data Fig. S1) also separates euphyllophytes from lycophytes and, within the latter, it supports as clades the lycopsids and Barinophytaceae + *Bathurstia*. Moreover, the Bayesian all-compat tree (Fig. 5B) supports a Sawdoniaceae clade with the same membership as that supported by parsimony. It is not unusual for Bayesian analyses to obtain low resolution with morphology-only datasets (e.g. O’Reilly and Donoghue, 2018), like those addressing fossil groups (e.g. Toledo *et al*., 2021). For this reason and because all the previous studies of zosterophyll phylogeny were conducted under parsimony, we limit our discussions below to the results of the parsimony analyses.

Notably, exclusion of only two ‘wildcard’ terminals (*Gosferia* and *Gutzeitia*) leads to a fully resolved tree (Fig. 5A) and the alternative placements of these two terminals show that only *Gosferia* is responsible for lowering phylogenetic resolution (see Supplementary Data Discussion). Thus, overall our dataset supports an unexpected level of phylogenetic resolution for the most comprehensive sampling of zosterophylls and allied taxa to date.

#### Comparisons with previous analyses (see extended considerations in the Supplementary Data Discussion)

**Fig. 10.**
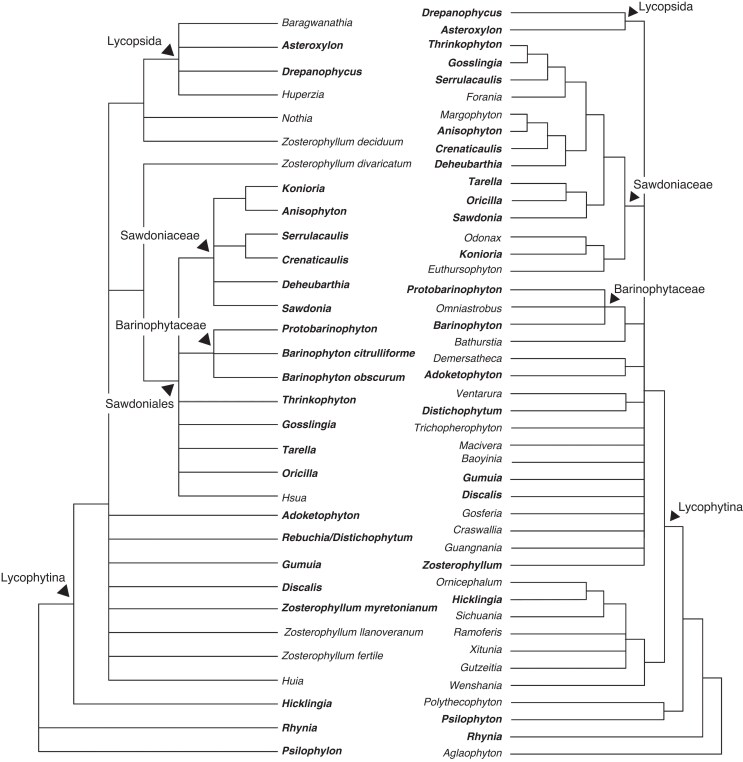
Comparison of topology between strict consensus topologies supported by our FTS analysis (right) and analysis 5.1 by Kenrick and Crane (1997; Fig. 5.25) (left; clade names same as in the original figure). Taxa in boldface are those common to the two analyses.

Placement of the lycopsids nested within a broader clade that includes all the zosterophylls (termed here the Lycophytina, following Kenrick and Crane (1997)), supported by our results, is in accord with previous results or predictions made by Gensel (1992), Kenrick and Crane (1997) and Crepet and Niklas (2019). The placement of Barinophytaceae nested within Lycophytina is also congruent with the results of Kenrick and Crane (1997) (Fig. 10). These results contrast those of Hao and Xue (2013), in which zosterophylls (Zosterophyllopsida) form a clade that excludes the lycopsids and the barinophytes. The Sawdoniaceae clade supported in our analyses includes all the taxa that are included in the Sawdoniaceae of Kenrick and Crane (1997), as well as all the zosterophyll taxa recovered by Kenrick and Crane in their Sawdoniales clade (Fig. 10) and those included in the crown clade supported in the analyses of Nibbelink and Tomescu (2022). Our results are also consistent with the polyphyletic status of genus *Zosterophyllum* demonstrated by Kenrick and Crane (1997): *Demersatheca*, *Gutzeitia* and *Ornicephalum*, three genera based on species placed initially in *Zosterophyllum* (Li and Edwards, 1996; Snigirevsky *et al*., 2007; Edwards and Li, 2018a), are scattered across the trees in our analyses (Figs 4 and 5A), instead of being grouped together.

The main difference between our results and those of Kenrick and Crane is that whereas the latter allow for sister-group relationship between Barinophytaceae and the remaining Sawdoniales (all of which are included in our Sawdoniaceae), with the lycopsids only distantly related to either of the two clades, our analyses provide strongest support for lycopsids sister to the Sawdoniaceae and forming together a clade that is sister to the Barinophytaceae. These differences may derive from (1) differences in the characters used; (2) differences in the scoring of some characters due to different interpretation of fossil features or additional data from post-1997 studies; or (3) the inclusion of additional taxa that may alter the polarization of characters.

#### An updated perspective on relationships among lycophytes

Our parsimony analyses support lycophyte monophyly with a basal dichotomy into two major clades, the larger of which (LBLC) includes a Sawdoniaceae clade, a Barinophytaceae + *Bathurstia* clade and a lycopsid clade (Fig. 5A). We recovered Sawdoniaceae and lycopsids as sister groups, together forming a clade that is sister to Barinophytaceae + *Bathurstia*; the remaining zosterophylls form a grade paraphyletic to the Barinophytaceae + *Bathurstia* and Sawdoniaceae + lycopsids clades. This pattern of relationships is generally similar to Kenrick and Crane’s (1997) results and supports some of their implications, despite a different taxon sampling and characters.

In a more conservative interpretation, taking into account the Bayesian analyses, recovery of the same Sawdoniaceae clade and its sister group relationship to the lycopsids as in the parsimony analyses, despite moderate to low congruence in topologies supported by the two tree-search methods, provides the strongest support to date for this pattern of relationships. Even in a most conservative perspective, independent of the topologies supported by each of the two tree-search methods, we can still identify a relative order (cohorts) of phylogenetic divergence that provides a rough measure of how derived a taxon is. While not all the taxa can be assessed from this perspective (due to differing degrees of stability), the most derived taxa include the members of the Sawdoniaceae, the lycopsids and the Barinophytaceae and *Bathurstia*, whereas *Craswallia*, *Baoyinia*, *Distichophytum*, *Macivera*, *Sichuania*, *Discalis* and *Gumuia* are the least derived taxa.

### Zosterophylls and lycophytes: taxonomy in the light of phylogenetic relationships

#### Previous taxonomic schemes (see extended version in Supplementary Data Discussion)

In summary, the taxonomic schemes proposed previously for lycophytes support, explicitly or implicitly, one of two major patterns of relationships. In one of them, zosterophylls form or are part of a grade paraphyletic to the lycopsids. This pattern of relationships is implied in Banks’ (1968) evolutionary hypothesis, wherein lycopsids (termed Lycophytina) arise from among a zosterophyll (Zosterophyllophytina) plexus, and is supported by the results of Gensel (1992) and Nibbelink and Tomescu (2022) (Table 3). Similar but not entirely congruent with this pattern of relationships, Kenrick and Crane’s (1997) Lycophytina clade consists of a grade paraphyletic to the lycopsids (Lycopsida); within this grade, traditionally recognized zosterophylls are polyphyletic and only a subset of them form a clade (Zosterophyllopsida) (Table 3). In contrast to this pattern, the analyses of Hao and Xue (2013) do not recover the lycophytes as a clade; instead, they support a zosterophyll clade (Zosterophyllopsida) sister to a clade that includes the lycopsids (Lycopsida) and the euphyllophyte, each forming separate clades (Table 3). A zosterophyll clade is also implied by Gerrienne *et al*.’s (2016) sister-group relationship between the Zosterophyllopsida and Lycopsida lineages within a more inclusive Lycophytina clade.

**Table 3.** Taxonomy and relationships of zosterophylls and lycopsids in this and previous studies.

	Zosterophylls	Zosterophyll membership included	Lycopsids	Zosterophyll–lycopsid relationships
Banks, 1968, 1975	Subdivision Zosterophyllophytina	6 genera	Subdivision Lycophytina	Lycopsids hypothesized as evolved from zosterophyll ancestor (zosterophylls form grade paraphyletic to lycopsid clade)
Gensel, 1992	Equated with subdivision Zosterophyllophytina Banks	18 genera (7 included in phylogeny)	Lycopsids	Zosterophylls form grade paraphyletic to lycopsid clade
Kenrick and Crane, 1997	Plesion Zosterophyllopsida (= Zosterophyllaceae) clade + Plesion *Z. myretonianum* + other taxa; relationships poorly resolved	23 species included in phylogeny (12 in Zosterophyllopsida, along with Barinophytaceae)	Class Lycopsida (= Lycophyceae)	Zosterophylls (including barinophytes, *Nothia*, *Hsua* and *Huia*) form grade paraphyletic to lycopsid clade; together included in subdivision Lycophytina (clade)
Hao and Xue, 2013	Zosterophyllopsida clade	8 species (6 genera) included in phylogeny	Lycopsida clade	Zosterophylls form with *Adoketophyton* a clade that is sister to a clade that includes lycopsid clade and euphyllophyte clade
Gerrienne *et al*., 2016	Zosterophyllopsida lineage (i.e. implied clade)	Not listed	Lycopsida lineage (i.e. implied clade)	Not explicitly discussed; zosterophylls and lycopsids are considered separate lineages within clade Lycophytina *sensu* Kenrick and Crane (1997)
Nibbelink and Tomescu, 2022	Zosterophylls	19 species (18 genera) included in phylogeny	Lycopsids	Zosterophylls form grade paraphyletic to lycopsids
This study	Zosterophylls	33 genera included in phylogeny	Lycopsids (= class Lycopsida)	Zosterophylls (including barinophytes) form grade paraphyletic to lycopsids; together included in subdivision Lycophytina (clade)

#### Updated perspective

Consistent with the results of Kenrick and Crane (1997) and Nibbelink and Tomescu (2022), our analyses support a Lycophytina clade wherein traditionally recognized zosterophylls are polyphyletic and part of a grade (which also includes the barinophytes + *Bathurstia* clade) that is paraphyletic to the lycopsid clade (Table 3). This reinforces the position of lycopsids as crown-group Lycophytina, with zosterophylls defining the stem of the clade. The synapomorphies of Lycophytina – lateral bilaterally symmetrical sporangia with dehiscence along the distal margin – fit among those listed by Kenrick and Crane (1997) for the clade. Two more of their synapomorphies, the reniform and isovalvate morphology of sporangia, not supported by our results, are predicted as ancestral in Lycophytina by our ancestral character stare reconstruction (Table 2, Fig. 9). In contrast, exarch xylem maturation, short-stalked sporangia and cellular thickening of the sporangium dehiscence line, also listed by Kenrick and Crane as Lycophytina synapomorphies, are not supported by our results.

The Sawdoniaceae, a well supported clade, group almost all the zosterophylls that were grouped together in previous analyses. Our Sawdoniaceae share with Kenrick and Crane’s (1997) Sawdoniaceae the spinescent emergences as a synapomorphy. However, in contrast to their Sawdoniaceae, ours are defined by three other synapomorphies and include more zosterophyll genera, overlapping much more closely with Kenrick and Crane's Sawdoniales (Fig. 10). However, Kenrick and Crane's Sawdoniales clade also includes the barinophytes and is supported by three characters that are not synapomorphies for any of the clades in our analyses. Nevertheless, two other synapomorphies of our Sawdoniaceae – circinate axis tips and xylem elliptical in cross-section – are synapomorphies for Kenrick and Crane's Zosterophyllopsida, a clade consisting of their Sawdoniales plus *Zosterophyllum divaricatum* (Fig. 10). Additionally, another synapomorphy of Kenrick and Crane's Zosterophyllopsida – sporangia in two rows – is predicted as ancestral for our Sawdoniaceae clade (Table 2, Fig. 8 right).

In a nomenclatural perspective, we note that our Sawdoniaceae clade is more similar in its zosterophyll membership to Kenrick and Crane’s (1997) Sawdoniales than to their Sawdoniaceae, and it is more similar in synapomorphies to their even more inclusive Zosterophyllopsida. However, our Sawdoniaceae cannot be equated to (and designated as) Sawdoniales or Zosterophyllopsida because it differs from Kenrick and Crane's two clades in excluding the barinophytes. Additionally, any clade that includes Sawdoniaceae and barinophytes in our phylogeny also includes the lycopsids, which are excluded from Kenrick and Crane's Sawdoniales and Zosterophyllopsida. Thus, given the relationships supported by our analysis, the latter two taxa have no equivalent in our phylogeny and our use of Sawdoniaceae is justified.

### Evolutionary implications

#### Tempo of evolution (see additional considerations on lycophyte fossils not included our analysis in the Supplementary Data Discussion)

In a phylogenetic context, the lower limit of the stratigraphic range of a terminal provides a minimum age for the cladogenetic event that gave rise to the least inclusive clade of which that terminal is a member. Applied to our best stratigraphic fit tree (Fig. 6), this line of reasoning allows the reconstruction of evolutionary tempo in the lycophyte clade, as supported by our analyses.

The age and position of *Macivera* suggest that an initial Silurian radiation gave rise to all the major lycophyte clades during the early Ludlow (Gorstian) or possibly earlier. However, ancestral lineages in each of these clades may have undergone little cladogenesis until close to the end of the Silurian, throughout the late Ludlow (Ludfordian) and the Pridoli, as indicated by the Lochkovian age of high-divergent terminals in the SBL clade, in the clade sister to *Macivera*, and also to some extent in the clade that includes *Z. myretonianum* (Fig. 6). The latest-Silurian (Pridoli) gap in the zosterophyll fossil record means that we do not know what the ancestors of these major clades looked like, other than what we can infer about them from reconstructed ancestral character states (see below).

The stratigraphic distribution of terminals also supports a major radiation that gave rise to much of the known genus-level diversity in all the lycophyte clades before the Devonian, in the ∼6-million-year interval between the end of the Gorstian and the Lochkovian. In this radiation, the ancestor of the lycopsid clade diverged from its zosterophyll sister group (most likely the ancestor of Sawdoniaceae) and the *Bathurstia* + Barinophytaceae clade diverged from the Sawdoniaceae + lycopsids clade (Fig. 6). This end-Silurian diversification episode is followed by continued cladogenesis within the lycophyte sub-clades (probably including the early barinophyte radiation) during the first ∼8 million years of the Devonian (Lochkovian), with the Pragian witnessing diversification only in a few minor clades and virtually no diversification after the Pragian, ∼408 Ma.

#### Cross-validating the best stratigraphic fit topology and cohorts of phylogenetic divergence

Taxa placed in the lowest diverging cohort (Figs 5 and 7), which should have arisen in the earliest pulses of diversification, are, indeed, products of early, pre-Devonian (Silurian) radiations. A good example of such congruence between stratigraphic position and membership in the lowest diverging cohort are the taxa of the SBLC (Figs 5 and 6). There are also exceptions: *Craswallia*, *Discalis*, *Gumuia* and *Distichophytum*, which may have arisen from smaller diversification bursts in the Lochkovian or earliest Pragian.

Conversely, most taxa of the highest-diverging cohort and the highest-diverging group of the median cohort are products of post-Silurian (Lochkovian and Pragian) diversification pulses. Here, the exceptions are the lycopsid clade and the *Deheubarthia–Crenaticaulis–Anisophyton–Margophyton* clade. The latter is intriguing because it is one of the most derived clades of the Sawdoniaceae and, as such, it is also the highest-diverging group among the high-diverging cohort. However, recognizing that this incongruence between phylogenetic topology and cohorts of divergence is due exclusively to the deep stratigraphic range of the most derived terminal in that clade, *Margophyton* (Fig. 6), brings the latter into focus.

The case of *Margophyton* merits discussion because it is emblematic for the influence that the accuracy of taxonomic and stratigraphic data of a single terminal can have on congruence between phylogenetic topology and cohorts of divergence. *Margophyton* was established in 1981 based on specimens from Russian localities (Zakharova, 1981). The stratigraphic constraints on layers containing *Margophyton* were relatively imprecise – Pragian–Emsian in all but one of the fossil localities, which was even less stringently dated as Lower Devonian. Compounding the stratigraphic imprecision, *Margophyton* was defined by Zakharova as a new genus for transferring fertile specimens that would have otherwise been assigned to *Psilophyton goldschmidtii* (= *P. burnotense*), a form-species based on vegetative material of morphologically simple early tracheophytes. Remains attributed to *P. goldschmidtii* probably represent more than one natural species and possibly multiple higher-order taxa, moreover. *P. goldschmidtii* has been reported from multiple localities around the world that span the entire Lower Devonian (Zakharova, 1981). Despite these caveats, Zakharova advocated inclusion of all material previously attributed to *P. goldschmidtii* material in *Margophyton*, without critical assessment of all that material. As a result, some European specimens that could be characterized as *Margophyton* were found with terminal sporangia typical of the euphyllophytes, such as *Psilophyton* (Schweitzer, 1989; Capel *et al*., 2022, 2024), implying that the European material is distinct from the Russian one. Thus, the taxonomic imprecision introduced by treating all *P. goldschmidtii* specimens as *Margophyton* inflates the stratigraphic range of the latter, so the range of *Margophyton* used in our analysis is probably much broader than the real age of the taxon and may not have reached as deeply as the basal Devonian. For instance, if *Margophyton* is limited to Zakharova's Russian material and its age is constrained to Pragian–Emsian (the age of most of that material), this would make the minimum age of the *Deheubarthia* node no older than late Lochkovian, consistent with placement of that clade in the highest-diverging cohort (Fig. 6). With that, the minimum age of the entire Sawdoniaceae clade, lycopsids, *Bathurstia* + Barinophytaceae and the *Trichopherophyton* node would also be late Lochkovian and consistent with their placement in the respective cohorts of phylogenetic divergence.

The predictive power of the best stratigraphic fit tree and the separation of taxa into cohorts of phylogenetic divergence and, consequently, the congruence between their results are affected by multiple factors: (1) the incompleteness of the fossil record (e.g. the Pridoli gap) and, related to it, the fact that fossils only provide minimum ages of cladogenetic events; (2) inconsistencies among terminals in the stringency of stratigraphic constraints on their age, sometimes associated with (3) taxonomic imprecision due to old or unrevised treatments of taxa (e.g. *Margophyton*); (4) the level of congruence between the topologies supported by the parsimony versus Bayesian analyses; and (5) the relatively arbitrary way in which the cohorts of phylogenetic divergence are separated. Given all these factors potentially biasing the analyses, the level of congruence between the evolutionary tempo predicted by the parsimony tree and the membership of the phylogenetic divergence cohorts is remarkable.

Our results suggest that approaches like the one presented here, which quantify relative sequences of phylogenetic divergence based on comparisons of incongruent topologies supported by different methods of phylogenetic inference – e.g. maximum parsimony and Bayesian – and independent of the specific topologies, may hold value. Especially in cases where different methods of inference yield highly incongruent topologies, such analyses can at least identify common denominators, such as our cohorts of phylogenetic divergence. So, we encourage further exploration of, and experimentation with, such approaches, both in terms of the metrics used to order sequences of divergence and of the criteria used to separate cohorts, alongside continued updating and refining of the ages and taxonomy of fossil taxa.

#### Mode of evolution (see additional considerations on apical circination, surface emergences and sporangium-bearing appendages in the Supplementary Data Discussion)

Aspects of evolutionary mode (groups in which certain characters evolved and how or whether they changed subsequently within those groups) can be reconstructed from the distribution of characters on phylogenetic trees. Specifically, such inferences are informed by the distribution of character states among terminals, combined with identification of synapomorphic character states and reconstruction of ancestral character states. In a conservative approach, we examine morphological evolution in terms of patterns (and not in terms of processes) based on the strict consensus tree of the parsimony analysis with full taxon sampling (Figs 4, 8 and 9).

The common ancestor of Lycophytina is reconstructed as a plant with simple axes lacking apical circination that bore laterally sporangia with irregular (i.e. not rowed) arrangement. The sporangia were bilaterally symmetrical, reniform, bivalvate with equal valves (isovalvate) and dehisced along their distal margin. The isovalvate sporangia are broadly conserved within the clade, with exceptions in *Ventarura* (sporangia with larger abaxial valves) and the sporangia with smaller abaxial valves evolved independently in some members of the SBL and LBL clades (including some of the Sawdoniaceae).

The ancestor of the SBL clade conserved the irregular arrangement of sporangia, typically distributed on multiple orders of branching; however, their ancestrally reniform morphology, conserved in *Wenshania*, evolved to more spherical shapes in the rest of the clade. The LBL clade, typically characterized by K-/H-branching and 3-D branching, evolved two-rowed sporangia. Within this clade, the ancestral reniform sporangial morphology evolved to other shapes outside the Sawdoniaceae and lycopsid clades, and the two-rowed sporangial arrangement reversed to irregular sporangiotaxis or changed to a taxis not strictly two-rowed, in some taxa.

The *Bathurstia* + Barinophytaceae ancestor conserved the regular, two-rowed arrangement of sporangia plesiomorphic in the LBL clade, but the barinophytes diverged from the ancestral reniform sporangium morphology present in *Bathurstia*, evolving lenticular sporangia held on specialized appendages.

The Sawdoniaceae ancestor evolved apical circination of axes and emergences on the axes (subsequently lost in some members of the clade). This plant probably had xylem with elliptical cross-sectional shape and sporangia distributed on multiple orders of branching. The ancestrally two-rowed sporangia were not conserved in the clade, most members reversing to irregular sporangiotaxis or evolving differently rowed sporangia, so that the two-rowed sporangia of relatively highly derived *Odonax* probably represents independent evolution of the condition.

The evolution of specific zosterophyll branching morphologies merits consideration here. Branching morphologies present among lycophytes and not encountered in other early tracheophytes include the feature termed here ‘branches parallel to subtending axis’ and the K-/H-branching. The former is a branching morphology wherein branches curve apically close to their base to run more or less parallel with the subtending axis (e.g. *Oricilla*; Gensel, 1982). This morphology is a putative synapomorphy for the least inclusive clade that contains *Z. myretonianum* (Figs 5A and 6), although missing data (*Craswallia*, *Gumuia*, *Discalis* and *Gosferia*) preclude its unequivocal scoring throughout the clade. This branching morphology probably evolved independently in other members of the LBL clade: the *Distichophytum* + *Ventarura* clade and members of the Sawdoniaceae (*Odonax*, *Sawdonia*, *Oricilla* and *Forania*).

K-/H-branching (reviewed by Matsunaga *et al*., 2017) is typically seen in rhizomatous portions of the plants. In it, a lateral branch dichotomizes apically close to its base forming two branches with different orientations: one grows in the same direction as the main axis, the other grows in the opposite direction; the latter usually takes on a rooting function. K-/H-branching has been documented in zosterophylls and lycopsids scattered all across the LBLC that are not directly related (*Z. myretonianum*, *Adoketophyton*, *Bathurstia*, *Odonax*, *Sawdonia*, *Tarella*, *Drepanophycus* and possibly *Asteroxylon*; see Hetherington *et al*., 2021 for the last of these), which would indicate that it evolved independently several times in this clade. However, the presence of K-/H-branching can be documented unequivocally in a taxon only under particular circumstances: (1) when many specimens representing the entire body plan are available; or (2) if exceptional specimens fortuitously preserve it. Additionally, (3) the rhizomatous portions of the plants that typically have K-/H-branching are the least likely to be preserved in fossil assemblages that are not preserved *in situ*; and (4) most lycophyte fossils are not known from such fossil assemblages. As a result, the presence/absence of K-/H-branching cannot be documented unequivocally in many of the taxa included in our analysis. Thus, we cannot rule out that this branching morphology may have evolved in the common ancestor of a clade that includes lycopsids and (at least some) zosterophylls, the more so as echoes of it may well be present in some living lycopsids (Matsunaga *et al*., 2017; Fujinami *et al*., 2021).

## Conclusions

Zosterophylls, the most diverse tracheophytes during the first part of the Early Devonian, are thought to have given rise to the lycopsid lineage. However, phylogenetic relationships among lycophytes – i.e. among and between zosterophylls and lycopsids – have remained the subject of competing hypotheses. Our analyses including the most comprehensively sampled zosterophyll dataset to date, under parsimony, obtained improved resolution compared with previous analyses, allowing us to address different aspects of evolution within the lycophyte clade. We also introduce new methods of: (1) quantifying taxon stability when dealing with incongruent topologies generated by different inference methods based on the same dataset; and (2) drawing general inferences on phylogenetic succession, i.e. organizing taxa based on their position of phylogenetic divergence with respect to the stem versus crown of a phylogenetic tree; and we demonstrate the applicability of these methods.

We found support for zosterophylls forming a grade paraphyletic to the lycopsids, which indicates that zosterophylls can be regarded as a diverse, extinct stem-group of the lycophyte clade. Within the zosterophyll grade, a few main clades have moderate support, whereas a larger Sawdoniaceae clade is consistently recovered. Barinophytes, forming a clade, are nested within the zosterophyll grade. We also find moderate support for Sawdoniaceae sister to the lycopsids, with the Sawdoniaceae–lycopsids clade sister to a clade consisting mainly of barinophytes.

The phylogenetic relationships supported by our analyses, along with the stratigraphic distribution of taxa and the phylogenetic distribution of their documented or inferred characters (reconstructed ancestral character states), shed light on the tempo and mode of zosterophyll and lycophyte evolution. Our results support a single major diversification phase of zosterophylls (which also gave rise to the ancestral lycophyte) during the late Silurian. This phase was followed by continued cladogenesis among zosterophylls during the Lochkovian, scarce cladogenesis in the Pragian, and virtually no diversification afterwards. Lycophytes (Lycophytina) are ancestrally defined by sporangia attached laterally on axes, possessing bilateral symmetry, bivalvate and opening along their distal margin. Lycophytina comprises two clades, one characterized by irregular distribution of sporangia along axes and the other (including the majority of zosterophylls along with lycopsids and barinophytes) hosting members with reniform sporangia arranged in rows and possessing 3-D branching architectures and K-/H-branching in the rhizomatous portions.

A previous study by Capel *et al*. (2023*a*) suggested preferential distribution of zosterophylls in equatorial areas. While integrating the evolutionary information contained in phylogenies with the geographical distribution of taxa falls outside the scope of this study, future studies addressing this aspect based on our expanded dataset will add another layer to our understanding of evolutionary tempo and mode among zosterophylls and lycophytes. This is especially relevant since zosterophylls are found all around the world and the Devonian is thought to have witnessed significant tectonic plate movements (Scotese, 2016).

## Supplementary Material

mcaf146_Supplementary_Data

## SUPPLEMENTARY DATA

Supplementary data are available at Annals of Botany online and consist of the following. Table S1: indices and tests of stratigraphic fit for the six MP trees recovered in the FTS analysis. Methods: taxa and characters used in the phylogenetic analyses. Methods S1: list of taxa included in the analyses. Methods S2: list of taxa not included in the analyses. Methods S3: characters used in the phylogenetic analyses. Methods S4: example of calculation of taxon stability and separation of cohorts of phylogenetic divergence. Figure S1: majority rule consensus tree of Bayesian analysis with full taxon sampling. Discussion: The pattern of relationships among lycophytes: past and present. Unstable taxa. Comparisons with previous analyses. Zosterophylls and lycophytes: taxonomy in the light of phylogenetic relationships. Previous taxonomic schemes. Evolutionary implications. Tempo of evolution. Mode of evolution. References. Data Sheet S1: character scoring for the phylogenetic matrix (Excel spreadsheet). Data Sheet S2: phylogenetic matrix (Nexus file). Data Sheet S3: occurrences of taxa included in the analyses and their ages.
