# Supplementary material for: Re-evaluating the phylogenetic relationships of zosterophylls with a comprehensively sampled dataset and a combination of traditional and new alternative methods
Source: Ann Bot. 2025 Jul 10;137(6):1624–45. doi: 10.1093/aob/mcaf146 (PMC13274996; doi:10.1093/aob/mcaf146)
Supplement: mcaf146_Supplementary_Data [file mcaf146_supplementary_data.zip › ClaisseEtAl2024_supplinfo_R2.docx]

**Supplementary Material**

**Reevaluating the phylogenetic relationships of zosterophylls with a comprehensively sampled dataset and a combination of traditional and new alternative methods**

## Pénélope Claisse*, Borja Cascales-Miñana, Eliott, Capel and Alexandru M.F. Tomescu*

*For correspondence. Email penelope.claisse@hotmail.com and [mihai@humboldt.edu](mailto:mihai@humboldt.edu)

**Supplementary Table 1.** Indices and tests of stratigraphic fit for the six most parsimonious trees recovered in the FTS analysis.

**Supplementary Methods.** Taxa and characters used in the phylogenetic analyses.

1. List of taxa included in the analyses

2. List of taxa not included in the analyses

3. Characters used in the phylogenetic analyses

4. Example of calculation of taxon stability and separation of cohorts of phylogenetic divergence.

**Supplementary Figure 1.** Majority rule consensus tree of Bayesian analysis with full taxon sampling.

**Supplementary Discussion**

# The pattern of relationships among lycophytes: past and present

# Unstable taxa.

# Comparisons with previous analyses

## Zosterophylls and lycophytes: taxonomy in the light of phylogenetic relationships

# Previous taxonomic schemes

# Evolutionary implications

# Tempo of evolution

# Mode of evolution

# **References**

**Supplementary Table 1.** Indices and tests of stratigraphic fit for the six most parsimonious trees recovered in the FTS analysis. Abbreviations: SRL = Simple Range Length; MIG = Minimum Implied Gap, with GMax and GMin respectively the maximum and minimum gaps within the tree; SCI = Stratigraphic Congruence Index; RCI = Relative Completeness Index; GER = Gap Excess Ratio; Est.p.[INDEX] and p.Wills are the significance tests; all tests and indices are detailed in Bell and Lloyd (2015). Tree 6 (in boldface) has the overall best stratigraphic fit (BSF) and was used for the time-scaled phylogeny (Fig. 3).

|  | **SRL** | **MIG** | **GMax** | **GMin** | **SCI** | **RCI** | **GER** | **MSM*** | **est.p.SCI** | **est.p.RCI** | **est.p.GER** | **est.p.MSM*** | **GER*** | **GERt** | **p.Wills** |
| --- | --- | --- | --- | --- | --- | --- | --- | --- | --- | --- | --- | --- | --- | --- | --- |
| tree 1 | 407.2 | 343.7 | 753.9 | 48.2 | 0.71 | 15.59 | 0.58 | 0.140 | 0.24 | 0.95 | 0.95 | 0.95 | 0.151 | 0.00 | 1.00 |
| tree 2 | 407.2 | 339.8 | 753.9 | 48.2 | 0.71 | 16.55 | 0.59 | 0.142 | 0.24 | 0.70 | 0.70 | 0.70 | 0.487 | 0.37 | 0.685 |
| tree 3 | 407.2 | 339.8 | 753.9 | 48.2 | 0.71 | 16.55 | 0.59 | 0.142 | 0.24 | 0.70 | 0.70 | 0.70 | 0.315 | 0.37 | 0.849 |
| tree 4 | 407.2 | 337 | 753.9 | 48.2 | 0.69 | 17.24 | 0.59 | 0.143 | 0.92 | 0.37 | 0.37 | 0.37 | 0.648 | 0.63 | 0.513 |
| tree 5 | 407.2 | 335.9 | 753.9 | 48.2 | 0.71 | 17.51 | 0.59 | 0.143 | 0.24 | 0.25 | 0.25 | 0.26 | 0.829 | 0.74 | 0.352 |
| **tree 6** | **407.2** | **333.1** | **753.9** | **48.2** | **0.69** | **18.2** | **0.6** | **0.145** | **0.92** | **0.06** | **0.07** | **0.06** | **1.00** | **1.00** | **0.171** |

**Supplementary Methods.** Taxa and characters used in the phylogenetic analyses.

The taxon list includes both taxa included in the analyses (with references consulted for scoring the characters and the absolute age interval of each taxon) and taxa initially considered for inclusion but ultimately excluded (with justification for their exclusion).

The characters are accompanied (where necessary) by comments detailing their definitions or justifying the scoring approach and scoring decisions.

#

# **1.** List of taxa (in alphabetical order) included in the analyses and their absolute age intervals, based on the published chronostratigraphic ages of all occurrences of each taxon (see Supplementary Data Sheet 1 for occurrences taken into account in determining the stratigraphic range of each taxon; their corresponding absolute ages are based on Cohen *et al.*, 2012).

- - *Adoketophyton* (Li and Edwards, [1992](#_bookmark46); Hao *et al.*, [2003](#_bookmark27); Zhu *et al.*, [2011](#_bookmark69); Hao and Xue, [2013](#_bookmark29))

– Age: 419.2 to 407.6 Myr

- - *Aglaophyton* (Kidston and Lang, [1920a](#_bookmark40); Edwards, [1986](#_bookmark6); Remy and Hass, [1996](#_bookmark57))

– Age: 409.7 to 407.6 Myr

- - *Anisophyton* (Remy *et al.*, [1986](#_bookmark56); Gerrienne, [1991](#_bookmark21)a)

– Age: 407.6 to 393.3 Myr

- - *Asteroxylon* (Kidston and Lang, [1920b](#_bookmark41); Fairon, [1967](#_bookmark17); Kerp *et al.*, [2013](#_bookmark38); Hetherington *et al.*, [2021](#_bookmark30))

– Age: 409.7 to 407.6 Myr

- - *Barinophyton* (Arnold, [1939](#_bookmark0); Brauer, [1980](#_bookmark4), [1981](#_bookmark5))

– Age: 393.3 to 358.9 Myr

- - *Bathurstia* (Hueber, [1971a](#_bookmark31); Kotyk and Basinger, [2000](#_bookmark43))

– Age: 410.8 to 407.6 Myr

- - *Baoyinia* (Edwards and Li, [2018b](#_bookmark15))

– Age: 419.2 to 402.8 Myr

- - *Craswallia* (Morris and Edwards, [2014](#_bookmark51))

– Age: 416.4 to 410.8 Myr

- - *Crenaticaulis* (Banks and Davis, [1969](#_bookmark1))

– Age: 402.8 to 393.3 Myr

- - *Deheubarthia* (Edwards *et al.*, [1989](#_bookmark13))

– Age: 413.6 to 410.6 Myr

- - *Demersatheca* (Li and Edwards, [1996](#_bookmark48); Wang *et al.*, [2022](#_bookmark64))

– Age: 410.8 to 402.8 Myr

- - *Discalis* (Hao, [1989b](#_bookmark26))

– Age: 409.7 to 407.6 Myr

- - *Distichophytum* (Hueber, [1972](#_bookmark33); Schweitzer, [1987](#_bookmark59); Kotyk, [1998](#_bookmark42))

– Age: 410.8 to 393.3 Myr

- - *Drepanophycus* (Göppert, [1852](#_bookmark24); Li and Edwards, [1995](#_bookmark47))

– Age: 413.6 to 385.2 Myr

- - *Euthursophyton* (Mustafa, [1978](#_bookmark52))

– Age: 391.1 to 382.7 Myr

- - *Forania* (Jensen and Gensel, [2013](#_bookmark35))

– Age: 398 to 393.3 Myr

- - *Gosferia* (Gerrienne, [1999](#_bookmark23))

– Age: 407.6 to 402.8 Myr

- - *Gosslingia* (Edwards, [1970](#_bookmark8))

– Age: 413.6 to 407.6 Myr

- - *Guangnania* (Wang and Hao, [2002](#_bookmark62); Edwards *et al.*, [2016](#_bookmark11))

– Age: 410.8 to 402.8 Myr

- - *Gumuia* (Hao, [1989a](#_bookmark25))

– Age: 409.7 to 407.6 Myr

- - *Gutzeitia* (Snigirevsky *et al.*, [2007](#_bookmark61))

– Age: 377.4 to 372.2 Myr

- - *Hicklingia* (Kidston and Lang, [1924](#_bookmark39); Edwards, [1976](#_bookmark10))

– Age: 393.3 to 382.7 Myr

- - *Konioria* (Zdebska, [1982](#_bookmark68); Edwards *et al.*, [1989](#_bookmark13))
    - Age: 398 to 393.3 Myr
  - *Macivera* (Kotyk *et al.*, [2002](#_bookmark44))
    - Age: 425.6 to 423 Myr
  - *Margophyton* (Zakharova, [1981](#_bookmark67))

– Age: 413.6 to 393.3 Myr

- - *Odonax* (Gerrienne, [1996](#_bookmark22))

– Age: 407.6 to 402.8 Myr

- - *Omniastrobus* (Bonacorsi *et al.*, [2021](#_bookmark3))

– Age: 407.6 to 402.8 Myr

- - *Oricilla* (Gensel, [1982](#_bookmark18))

– Age: 398 to 393.3 Myr

- - *Ornicephalum* (Edwards and Li, [2018a](#_bookmark14))

– Age: 419.2 to 409.7 Myr

- - *Polythecophyton* (Hao *et al.*, [2001](#_bookmark60))

– Age: 409.7 to 407.6 Myr

- - *Protobarinophyton* (Brauer, [1981](#_bookmark5))

– Age: 413.6 to 358.9 Myr

- - *Psilophyton crenulatum* (Doran, [1980](#_bookmark7))

– Age: 409.7 to 387.7 Myr

- - *Ramoferis* (Hao and Xue, [2011](#_bookmark28), 2013)

– Age: 409.7 to 407.6 Myr

- - *Rhynia* (Kidston and Lang, [1920a](#_bookmark40); Lemoigne, [1970](#_bookmark45))

– Age: 409.7 to 407.6 Myr

- - *Sawdonia* (Hueber, [1971b](#_bookmark32); Gensel *et al.*, [1975](#_bookmark19); Rayner, [1983](#_bookmark55); Gensel and Berry, [2016](#_bookmark20))

– Age: 408.6 to 382.7 Myr

- - *Serrulacaulis* (Hueber and Banks, [1979](#_bookmark34); Berry and Edwards, [1994](#_bookmark2); Xu *et al.*, [2011](#_bookmark65))

– Age: 387.7 to 377.4 Myr

- - *Sichuania* (Edwards and Li, [2018b](#_bookmark15))

– Age: 419.2 to 409.7 Myr

- - *Tarella* (Edwards and Kenrick, [1986](#_bookmark12))

– Age: 410.8 to 409.7 Myr

- - *Thrinkophyton* (Kenrick and Edwards, [1988](#_bookmark36)a)

– Age: 410.8 to 409.7 Myr

- - *Trichopherophyton* (Lyon and Edwards, [1991](#_bookmark49))

– Age: 409.7 to 407.6 Myr

- - *Ventarura* (Powell *et al.*, [1999](#_bookmark54))

– Age: 409.7 to 407.6 My

- - *Wenshania* (Zhu and Kenrick, [19](#_bookmark9)99; Hao and Xue, [2013](#_bookmark28))

– Age: 409.7 to 407.6 Myr

- - *Xitunia* (Xue, [2009](#_bookmark66))

– Age: 416.4 to 410.8 Myr

- - *Zosterophyllum myretonianum* (Edwards, [1975](#_bookmark9))

– Age: 419.2 to 407.6 Myr

# **2.** List of taxa (in alphabetical order) not included in the analyses.

# Many of these taxa were initially described by the different authors as “incertae sedis”, although commonly viewed as zosterophylls. These taxa could be included in future analyses to test their taxonomic placement and phylogenetic relationships.

- - *Bracteophyton* (Wang and Hao, 2004)

– Age: 407.6 to 402.8 Myr

- - - *Bracteophyton* is more likely a Barinophytaceae than a zosterophyll. It was originally considered Tracheophyta incertae sedis because of the lack of anatomical information.
  - *Danziella* (Edwards, [2](#_bookmark9)006)

– Age: 407.6 to 410.8 Myr

- - - Edwards placed *Danziella* as a Plantae incertae sedis. The plant shows some similarities with zosterophylls but also with rhyniophytes, and Edwards pointed to the need for studying additional specimens to reach a more precise taxonomic placement.
  - *Faironella* (Gerrienne, [19](#_bookmark9)96a)

– Age: 407.6 to 402.8 Myr

- - - Originally described the taxon as incertae sedis because of its peculiar fertile units. After an observation of the specimen in the collection, we noticed a close morphological similarity with *Sawdonia*, but *Faironella* requires further study of its fertile parts.
  - *Gippslandites* (McSweeney *et al.*, [2](#_bookmark9)020)

– Age: 419.2 to 407.6 Myr

- - - Even if the authors assigned *Gippslandites* to the zosterophylls, their observations are based on a single, poorly preserved specimen. In particular, we had difficulty documenting with certainty characters linked to the dehiscence and attachement of the sporangia and thus, decided to exclude this taxon from the analyses.
  - *Hsua* (Li, [19](#_bookmark9)92)

– Age: 409.7 to 408.6 Myr

- - - The original description favors placement of *Hsua* among the Rhyniales. Although *Hsua* has reniform sporangia like many zosterophylls, the sporangia are borne singly and terminally on axes, unlike in zosterophylls.
  - *Huia* (Geng, [1985](#_bookmark9)b)

– Age: 410.8 to 402.8 Myr

- - - Huia was assigned by Geng to the Rhyniales.
  - *Jugumella* (Senkevitch, [1975](#_bookmark9))

– Age: 423 to 402.8 Myr

- - - Although subsequent studies have considered this genus as a probable zosterophyll, its original uncertain placement was the reason behind its exclusion from from our analyses.
  - *Kaulangiophyton* (Gensel *et al.*, [19](#_bookmark9)69)

– Age: 398 to 391.1 Myr

- - - *Kaulangiophyton* compares better to early lycopsids, such as *Asteroxylon* and *Drepanophycus*, which are not the main focus of this study, than to zosterophylls.
  - *Krithodeophyton* (Edwards, [19](#_bookmark9)68)

– Age: 410.8 to 402.8 Myr

- - - According to Edwards, *Krithodeophyton* aligns with the Barinophytaceae, which are not the main focus of this study, and possesses primary xylem with centrarch maturation, unlike the zosterophylls and lycopsids.
  - *Nothia* (Saadawy and Lacey, [197](#_bookmark9)9)

– Age: 409.7 to 407.6 Myr

- - - The systematic position of *Nothia* has been debated: originally attributed to the Subdivision Zosterophyllophytina by Banks (1968), *Nothia* has was later removed from the group (Banks, 1969), because of its mozaic of characters that combine ryhniophyte and zosterophyll features.
  - *Parazosterophyllum* (McSweeney *et al.*, [2](#_bookmark9)020)

– Age: 425.6 to 423 Myr

- - - This genus is based on a single, incompletely preserved specimen which does not allow unequivocal scoring of many of the characters in our matrix.

# **3.** Characters used in the phylogenetic analyses.

The characters are inspired from Kenrick and Crane ([1997](#_bookmark37)), Hao and Xue ([2013](#_bookmark29)) and Nibbelink and Tomescu ([2022](#_bookmark53)). However, due to the inclusion of numerous new taxa, a revision of characters definitions and character states was needed. Where applicable, numbers in brackets after the character name correspond to the character from previous publications that it is derived from: numbers in boldface - Kenrick and Crane ([1997](#_bookmark37)); italicized numbers - Hao and Xue ([2013](#_bookmark29)); numbers in boldface and italicized - Nibbelink and Tomescu ([2022](#_bookmark53)).

1. Rooting system: positively geotropic axes [New]

(0) Absence

(1) Presence

Many zosterophylls have been described with rhizomatous axes, which potentially bore rhizoids; these are scored “0” for this character. Lycopsids, on the other hand, have deeper rooting systems that include positively geotropic axes (and are scored “1”). Taxa in which the basal parts are not known were scored "?".

2. K-branching or H-branching ***[24]***

(0) Absence

(1) Presence

K- or H-branching refers to the particular branching pattern found in some zosterophylls and lycopsids, where two closely spaced branching points result in a morphology comparable to the letters K or H (see Matsunaga *et al.*, 2017). In many cases, one of the two resulting branches has a rooting function.

3. Branching type of erected axes **[5.1]** *[4]*

(0) Isotomous

(1) Pseudomonopodial

(2) Unbranched

The branching type refers to the relative size of axes produced at a branching point. In isotomous branching, one type of apical branching, the two branches are produced by an equal division of the apical meristem and have similar size (e.g., *Rynia* and *Aglaophyton*). The opposite of isotomous branching is anisotomous branching. When apical branching is strongly anisotomous, this results in an architecture similar to that produced by monopodial axillary branching; this type of anisotomous apical branching is termed pseudomonopodial (e.g., *Psilophyton*). Anisotomous branching is scored here the same ("1") whether it is pseudomonopodial or less strongly anisotonmous. Lastly, some plants, such as *Discalis* (Hao, 1989b; fig. 6), have erect aerial axes that are not branched (at least not in the specimens known to date), although the rhizomatous portions of the plant are branched; they are scored “2”. Additionaly, some descriptions document branching with "isotomous and pseudomonopodial dichotomies" in the same plant (e.g., Edwards *et al.*, 1989). In such cases, the taxon was scored "1", as pseudomonopodial branching, when present, can lead to additional architectural complexity, beyond that generated exclusively by isotomous branching. Nibbelink and Tomescu (2022) chose encode branching type in two characters, looking for the proximal and the distal branching (their char. 21 & 22), as inspired by the architecture of plants such as *Deheubarthia*, *Euthursophyton*, *Margophyton* or *Thrinkophyton*. However, here we decided to use a single character, since in many of the taxa included in our analysis the fragmentary material precludes identification of proximal vs distal portions of the plant. Thus, this choice of character construction reduces the amount of missing data in our dataset.

4. Branching pattern **[5.6]** *[5]*

(0) Planar

(1) Three-dimensional

(2) Branching absent

5. Branching angle [New]

(0) Narrow to wide

(1) Branches parallel to subtending axis

(2) Both types present

Because observed branching angles can reflect either natural branching angles or taphonomic results, branching angle was separated into only two types: one comprising branches that maintain more-or-less the same angle (narrow to wide, “0”) with respect to the main axes irrespective of distance from the branching point; and one in which the branch forms a narrow angle with the subtending axis at the base, but then curves apically close to its base and becomes more-or-less parallel to the axis (“1”), as seen in several taxa, e.g., *Distichophytum* (Hueber, 1972; plate I 1-3), *Forania* (Jensen and Gensel, 2013; fig. 2E), *Guangnania* (Edwards *et al.*, 2016; fig. 4B), *Odonax* (Gerrienne, 1996b; fig. 5), *Oricilla* (Gensel, 1982b; plate II 2-3), *Sawdonia* (Gensel and Berry, 2016; fig. 9A-C and Rayner, 1983; fig. 1a, 1d, 7), or *Zosterophyllum* (Edwards, 1975; fig. 15).

6. Circinate axis tip **[5.4]** *[6]* ***[26]***

(0) Absence

(1) Presence

Taxa in which axis tips are not known (not preserved) are scored "?".

7. Subaxillary branching **[5.3]**

(0) Absence

(1) Presence

The axes derived from subaxillary tubercles/buds are typically oriented in a direction perpendicular to the plane of the branches in whose subaxillary position the tubercle is located (see Matsunaga *et al.*, 2017). Subaxillary branches probably had a rooting function, similar to that of the rhizophores of *Selaginella*. The subaxillary tubercles (and corresponding branches, when developed) are sometimes erroneously referred to as axillary tubercles, despite their position *below* the branch axil. Taxa known only from fertile axes are scored "?" for this character.

8. Emergences; modified from **[5.5]** ***[16]***

(0) Absence

(1) Presence

Emergences are defined as unvascularized protrusions on the axes, sporangia or sporangial stalks. A number of characters listed below describe different features of the emergences. Those characters are scored “-“ (inapplicable) in taxa that lack emergences.

9. Emergences: localization (scored "-" in taxa that lack emergences) [New]

(0) On vegetative parts only

(1) On vegetative and fertile parts

(2) On fertile parts only (sporangium or sporangium stalk)

10. Emergences: biseriate arrangement (scored "-" in taxa that lack emergences) [New]

(0) Absence

(1) Presence

Some taxa show a regular organization in the distribution of their emergences, while other have emergences scattered without any defined pattern. Taxa with biseriate emergences include *Bathurstia* (in Kotyk and Basinger, 2000; fig. 10, fig. 18), *Crenaticaulis* (in Banks and Davis, 1969; fig. 12), *Forania* (in Jensen and Gensel, 2013; fig. 2C, fig. 7) and *Serrulacaulis* (in Berry and Edwards, 1994; fig. 1 and Hueber and Banks, 1979; plate I 2-4, fig. 1 and Xu *et al.*, 2011; fig. 1A).

11. Glandular structure on emergences (scored "-" in taxa that lack emergences) [New]

(0) Absence

(1) Presence

Some taxa have emergences with a rounded, flattened or darkened tip, which are interpreted as glandular structures or their remnants. These taxa include *Anisophyton* (in Remy *et al.*, 1986b; fig. 3, 4), *Discalis* (in Hao, 1989b; plate III 1, fig. 7), *Forania* (in Jensen and Gensel, 2013; fig. 3G-I) and *Sawdonia* (in Rayner, 1983; fig. 2b).

12. Sporangium emergences: position (scored "-" in taxa that lack emergences) [New]

(0) On abaxial valve

(1) On both valves

Among the taxa having sporangial emergences, this character distinguishes between those bearing emergences on the abaxial valve only, like *Odonax* (Gerrienne, 1996b; fig. 2), *Sawdonia* (Gensel and Berry, 2016; fig. 7) or *Xitunia*, and those bearing emergences on both valves, like *Discalis* or *Konioria*. No taxa are known that have emergences on the adaxial valve only.

13. Emergence: shape (scored "-" in taxa that lack emergences) [New]

(0) Conical

(1) Shelf-like

“Shelf-like” refers to ridge-like emergences that girdle the axes partially such as those seen in *Bathurstia* (Kotyk and Basinger, 2000; fig. 3, 4, 10A-B, 18).

14. Sporangium: position on sporophyte axis **[5.18]** *[26]*

(0) Terminal

(1) Lateral

This character separates the basal polysporangiophytes/tracheophytes such as *Rhynia* or *Aglaophyton*, as well as the trimerophytes, all of which have sporangia borne at the tips of axes, from zosterophylls, which have laterally attached sporangia.

15. Lateral sporangium: attachment: cauline (scored "-" in taxa with terminal sporangia) *[23]*

(0) On axis

(1) On specialized appendage

Sporangia attached directly on axes by their stalks are not associated with any specialized structures. Sporangia attached to specialized appendages include those of the barinophytes and of *Adoketophyton*.

16. Sporangium stalk size relative to sporangium size; modified from **[5.18]**

(0) Sessile

(1) Short

(2) Long

Sessile sporangia have no well-differentiated stalk. Taxa were scored as having long sporangial stalks (“2”) if the stalks were about the same size as (or larger than) the sporangia.

17. Sporangium stalk stoutness (based on stalk length/stalk width) (scored "-" in taxa with

sessile sporangia) ***[34]***

(0) Length/width < 1

(1) Length/width > 1

18. Vascular bundle supplying sporangium (scored "-" in taxa with sessile sporangia) [New]

(0) Absence

(1) Presence

19. Sporangial stalk orientation (scored "-" in taxa with terminal sporangia or sessile sporangia) [New]

(0) Stalk oriented at acute angle to subtending axis

(1) Stalk oriented at right angle to subtending axis

20. Sporangium: shape modified from **[5.13]** *[17]*

(0) more or less spherical

(1) more or less ellipsoidal

(2) more or less reniform

(3) lenticular vertically flattened

Although lycophytes, including zosterophylls, are traditionally recognized as bearing reniform sporangia, when all zosterophylls and allied taxa are considered, sporangium shape encompasses morphological diversity broader than just a reniform shape. The "lenticular vertically flattened" condition was defined for the barinophyte sporangia, which have ellipsoidal overall shape, but a particular orientation perpendicular to the axis to which there are atached.

21. Sporangium symmetry *[18]*

(0) sporangia roughly radially symmetrical only

(1) sporangia bilaterally symmetrical

Taxa scored as having radially symmetrical sporangia (“0”) include the trimerophytes, which have roughly fusiform sporangia, the rhyniopsids, and those taxa that have spherical sporangia (*Craswallia* and *Margophyton*).

22. Sporangium: dorsiventral flattening (scored “-“ in taxa with terminal sporangia); modified from ***[36]***

(0) Absence

(1) Presence

23. Sporangium dehiscence: type **[5.16]** *[20]* ***[38]***

(0) Along distal margin

(1) Along a lateral line

Zosterophylls and allied taxa have sporangia that dehisce along their distal margin, whereas those of trimerophytes dehiscence along a lateral line.

24. Sporangium dehiscence: thickening of sporangium wall along dehiscence line **[5.17]** *[21]* ***[39]***

(0) Absence

(1) Presence

Presence or absence of a thickening of the sporangial wall bordering the dehiscence line may signal certain specializations in dehiscence mechanisms (e.g., McSweeney *et al.*, 2020). The character was scored “0” in taxa where such a thickening is neither described, nor illustrated, but was scored “1” in some cases where such thickenings are illustrated, even if they were not described.

25. Sporangium: relative valve size (scored "-" in taxa with sporangia that do not dehisce along their distal margin an, in other words, are not bivalvate – char. 23) **[5.15] *[37]***

(0) Isovalvate

(1) Abaxial valve bigger than adaxial valve

(2) Abaxial valve smaller than adaxial valve

26. Sporangium: attitude **[5.19]**

(0) Lateral (pointing outwards)

(1) Pointing more or less apically

(2) Curved toward the axis

27. Sporangium: paired modified from *[27]*

(0) Absence

(1) Presence

This character aims to distinguish euphyllophytes, like *Psilophyton*, which have sporangia paired on one stalk/branch tip, from the other groups.

28. Sporangium: distribution by order of branching **[5.12]**

(0) Only in the highest order branches

(1) In the highest and the lower order branches

Lower order branches are here recognized as any axis segment between two successive dichotomies. Sporangia located only on the highest orders of branching, i.e., on terminal axis segments, suggest determinate growth of the fertile axis tips (which could have been produced periodically), whereas sporangia on lower orders of branching are consistent with indeterminate growth of fertile axes. Taxa known only by their apical fertile portions are scored "?".

29. Sporangium: grouping; modified from ***[27]***

(0) Absence

(1) Presence

Absence refers to cases where sporangia are scattered, i.e., distributed relatively randomly along the axes, or isolated (like in *Konioria*). Presence of grouping of sporangia refers to taxa where sporangia are distributed consistently in groups where the distance between successive sporangia is relatively constant. *Discalis* is scored "0" because although the sporangia are known only on terminal segments, it is unclear whether those segments would subsequently branch and, if so, whether the branches thus produced would also bear sporangia; additionally, the distance between successive sporangia varies relatively widely in *Discalis*. This character is not applicable for taxa with sporangia attached terminally on axes (char. 14; e.g., *Rhynia*, *Psilophyton*).

30. Sporangium grouping: distribution (scored "-" in taxa that lack consistent sporangial grouping – char. 29) ***[29]***

(0) Terminal, forming a strobilus

(1) Intercalary

Taxa with intercalary sporangial grouping are those with grouped sporangia on lower order branches (i.e., between successive axis branching points), such as *Crenaticaulis* (Banks and Davis, 1969; fig. 1).

31. Sporangium grouping: density in groups (scored "-" in taxa that lack consistent sporangial grouping – char. 29) ***[30]***

(0) Lax

(1) Compact

Compact grouping of sporangia was scored for taxa in which neighboring sporangia are partially overlapping, in contact with each other, or at the very least spaced out at a distance smaller than their size.

32. Strobilus: position (scored "-" in taxa that lack sporangial grouping – char 29 – and in taxa with intercalary sporangial grouping – char. 30) [New]

(0) On the main axis

(1) On lateral axes

Scored “?” in some taxa that are known from material that is too fragmentary (e.g., only from terminal fertile regions); scored “1” for taxa with isotomous branching that lack a well defined main axis.

33. Sporangium: distribution along axis **[5.10]** *[27]*

(0) Irregular

(1) Regular

This character aims to distinguish taxa with no apparent organization of sporangia along axes. Multiple descriptions fail to demonstrate unequivocally a helical arrangement of sporangia along axes (Edwards and Li, 2018a,b). For this reason, taxa described as possibly having helically arranged sporangia are scored “0”. The notable exception is for the lycopsids that have regularly distributed helical sporangia, and are thus scored as “1”. Other taxa scored as “1” display a rowed organization of sporangia.

34. Sporangia distributed in rows: number of rows (scored "-" in taxa with helically or irregularly distributed sporangia – char. 33); modified from **[5.10]**

(0) One or two

(1) More than two

(2) Always two

35. Sporangia distributed in rows: sporangiotaxis (scored "-" in taxa with helically or irregularly distributed sporangia – char. 33); modified from ***[31]***

(0) Opposite

(1) Alternate

36. Leaves; modified from *[7]*

(0) Absence

(1) Presence

This character distinguishes lycopsids, which bear true leaves (vascularized emergences with regular taxis, from zosterophylls.

37. Spore size **[5.20]**

(0) Homosporous

(1) Heterosporous (anisosporous)

The spore size condition present in a few of our taxa does not conform to typical heterospory, as the microspores are located in the same sporangium as the megaspores, a condition that is recognized as anisospory.

38. Protostele: cross-sectional shape; modified from **[4.14]** *[11]* ***[7]***

(0) Terete

(1) Elliptical

(2) Lobed

The lobed morphology of the protostele characterizes lycopsids.

39. Pattern of primary xylem maturation **[4.15]** *[12]* ***[2]***

(0) Xylem absent

(1) Centrarch

(2) Exarch

(3) No conspicuous pattern of maturation

(4) Mesarch

40. Metaxylem: secondary wall thickening pattern; modified from *[13]* ***[4]***

(0) Absent

(1) Scalariform

(2) Pitted

41. Metaxylem: pitting (scored "-" in taxa without pitted metaxylem – char. 40) *[14]* ***[6]***

(0) S-type

(1) C-type

(2) G-type

(3) P-type

The pitting types correspond to those defined by Edwards (2003).

42. Sterome [New]

(0) Absence

(1) Presence

A sterome is recognized as an outer layer of cells with thickened walls.

**4.** Example of calculation of taxon stability and separation of cohorts of phylogenetic divergence.


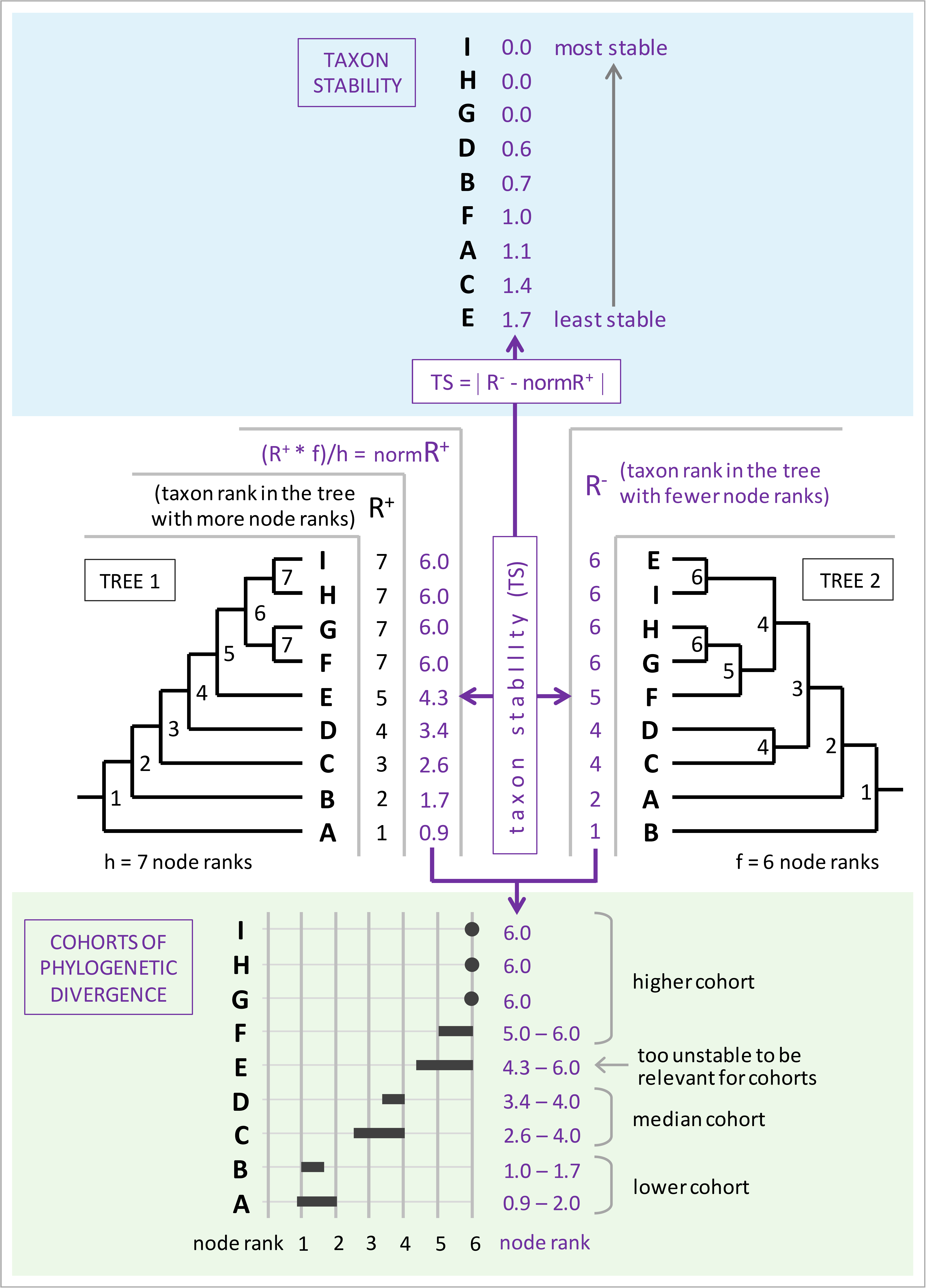


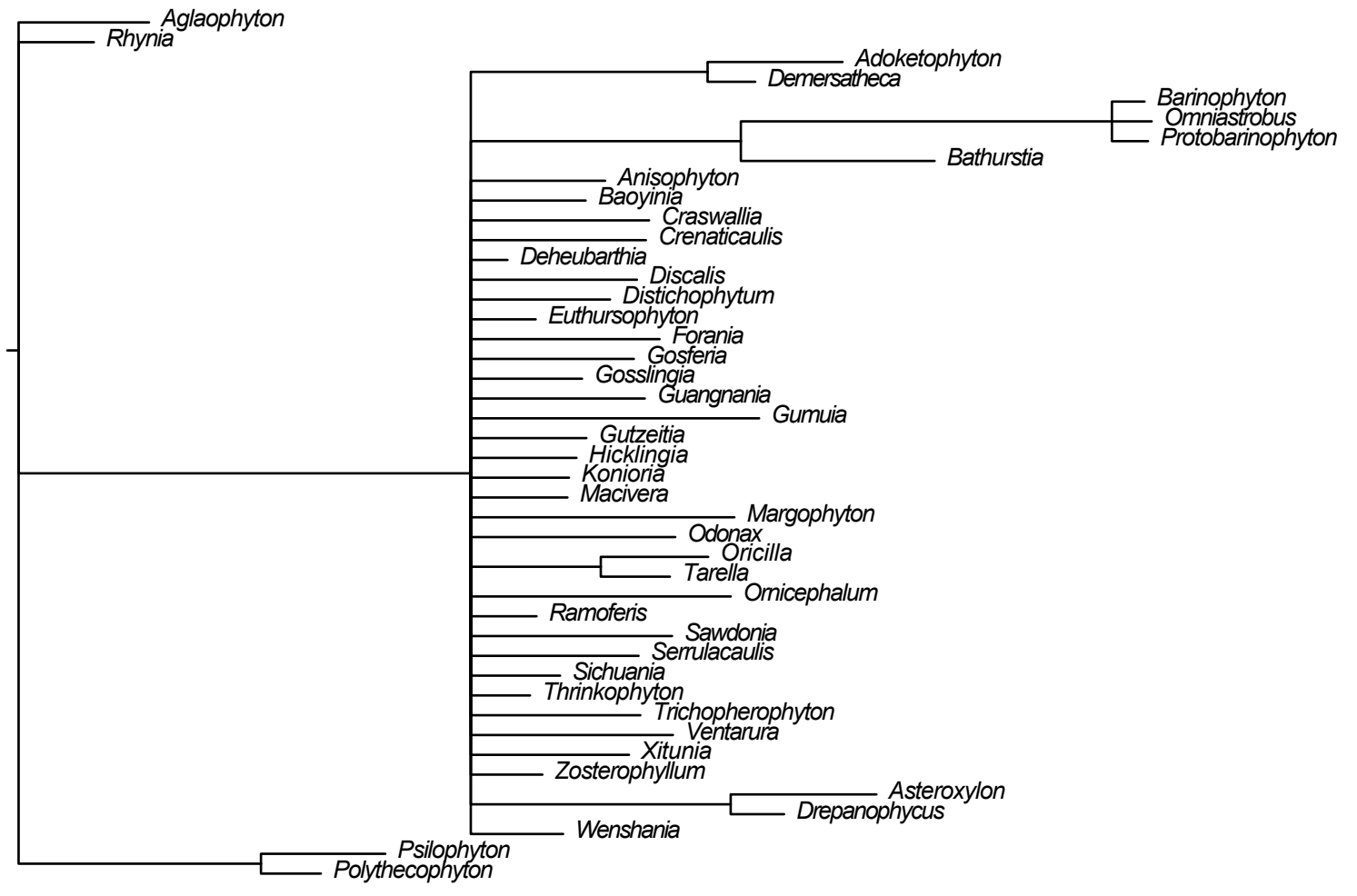


**Supplementary Figure 1.** Majority rule consensus tree of Bayesian analysis with full taxon sampling. The Bayesian analysis with *Gosferia* and *Gutzeitia* excluded (GGE) generated a similar majority rule consensus topology.

**Supplementary Discussion**

# *The pattern of relationships among lycophytes: past and present*

# *Unstable taxa*. Our taxon exclusion experiments demonstrated that *Gutzeitia* (Snigirevsky *et al.*, [2007](#_bookmark61)) and *Gosferia* (Gerrienne, 1991b, [1999](#_bookmark23)) lower the phylogenetic resolution obtainable from our dataset, despite not having the highest percentages of missing data among the taxa included. This suggests that their impact on resolution is more likely due to their specific combinations of characters.

# Among the six most parsimonious trees recovered in the FTS analysis, *Gutzeitia* oscillates between only two placements, both of them located in the small basal clade of Lycophytina (SBLC). Thus, rather than impacting resolution broadly across the phylogeny, *Gutzeitia* only affects relationships within the SBLC, where it is sister to *Xitunia* or to *Ramoferis*; the placement is supported in the best stratigraphic fit tree (Fig. 3). In contrast, *Gosferia* has a stronger impact on phylogenetic resolution, as it oscillates between alternative placements that are distant phylogenetically (neither of which is supported by multiple synapomorphies). One placement is as sister to the lycopsid clade, a relationship supported only by sporangial stalks that are longer than they are wide. The other placement is as sister to *Discalis*, supported by the length of sporangial stalks and by circination (which is only putatively present in the axes of *Gosferia*). The latter placement is supported in best stratigraphic fit tree (Fig. 3), which is also consistent with Gerrienne’s (1991) hypothesis on the taxonomic affinities of *Gosferia*.

# *Comparisons with previous analyses*. Placement of the lycopsids nested within a broader clade [termed here the Lycophytina, following Kenrick and Crane ([1997](#_bookmark44))] that includes all the zosterophylls, supported by our results, is in accord with previous results or predictions made by several authors, e.g., Gensel ([1992](#_bookmark27)), Kenrick and Crane ([1997](#_bookmark44)) and Crepet and Niklas ([2019](#_bookmark12)). The placement of the Barinophytaceae nested within Lycophytina (specifically, within a sub-clade of the latter) is also congruent with the results of Kenrick and Crane ([1997](#_bookmark44)) (Fig. 7). In contrast to this pattern of relationships, Hao and Xue ([2013](#_bookmark35)) have found support for a monophyletic clade of zosterophylls (Zosterophyllopsida) that excludes the lycopsids and the barinophytes, whether represented by *Barinophyton* or *Adoketophyton*. In their results, lycopsids and *Barinophyton* are recovered in a clade that is sister to the clade that includes the zosterophylls. *Adoketophyton*, recovered as sister to Zosterophyllopsida by Hao and Xue ([2013](#_bookmark35)), is nested deeply among zosterophylls (sister to *Demersatheca*) in our analysis, a placement not entirely incongruent with its position in Kenrick and Crane’s analysis.

The Sawdoniaceae clade supported in our analyses includes all the taxa included in the Sawdoniaceae of Kenrick and Crane ([1997](#_bookmark44)) (Fig. 7), as well as in the crown clade with a basal polytomy that was consistently recovered under different tree rooting and taxon sampling schemes by Nibbelink and Tomescu (2022). Beyond the overall similarity with Kenrick and Crane’s results, we note differences in the relationships of different taxa. We recover *Anisophyton* sister to *Margophyton* and not to *Konioria*, which is included in a different sub-clade. While *Crenaticaulis* and *Serrulacaulis* are recovered in sister clades within Sawdoniaceae, echoing their sister group relationship in Kenrick and Crane’s results, the former is more closely related to *Anisophyton* and *Margophyton* in our analyses. *Oricilla* and *Tarella*, included by Kenrick and Crane in an unresolved "Gosslingiaceae" group that is not directly related to the Sawdoniaceae, are nested in our analysis well within a clade that includes other members of Kenrick and Crane’s Sawdoniaceae, wherein they form a clade sister to *Sawdonia*.

The differences between our results and those of Kenrick and Crane ([1997](#_bookmark44)) may derive from (i) differences in the characters used; (ii) differences in the scoring of some characters due to different interpretation of fossil features or additional data generated on some of the taxa in post-1997 studies; or (iii) the inclusion of additional taxa that may alter the polarization of characters. Irrespective of what causes the differences, it is important to note that our Sawdoniaceae include all the zosterophyll taxa recovered by Kenrick and Crane in their Sawdoniales clade (Fig. 7). While Kenrick and Crane’s Sawdoniales also include the Barinophytaceae clade, the latter are part of the basal polytomy of the clade in the strict consensus tree. This pattern of relationships indicates that in some of the 18,500 most parsimonious trees found by Kenrick and Crane the Sawdoniales clades has a basal split between the Barinophytaceae and a clade whose members are all included in our Sawdoniaceae. Thus, the similarity between our results and those of Kenrick and Crane ([1997](#_bookmark44)) is deeper than suggested by differences between the placement of individual taxa. The main difference between the two analyses is that whereas Kenrick and Crane’s allows for sister-group relationship between Barinophytaceae and the remaining Sawdoniales (all of which are included in our Sawdoniaceae), with the lycopsids only distantly related to either of the two clades, our analyses provide strongest support for lycopsids sister to the Sawdoniaceae and forming a clade that is sister to the Barinophytaceae.

Another noteworthy result in the context of previous zosterophyll phylogenies is the placement of *Hicklingia* in the SBLC (Fig. 7). *Hicklingia* was recovered as sister to all the other lycophytes by Kenrick and Crane (1997), in apparent contrast to its placement in our analysis. However, when considering that all the other five species included in the SBLC with *Hicklingia* were discovered or revised taxonomically post-1996, the congruence between the two placements becomes obvious.

*Zosterophyllum*, the eponym genus of the group studied here and its first representative to be discovered (Penhallow, 1892), covers significant stratigraphic and geographic ranges among its ca. 31 species. Although our study does not directly address the relationships among all these species, our results are consistent with the polyphyletic status of genus *Zosterophyllum* demonstrated by Kenrick and Crane (1997). Specifically, *Demersatheca*, *Gutzeitia* and *Ornicephalum*, three genera based on species segregated from, or placed at some point in, *Zosterophyllum* (Li and Edwards, 1996; Snigirevsky *et al.*, 2007; Edwards and Li, 2018a), are scattered across the trees (Fig. 1, 2A) instead of being grouped together. Phylogenetic studies that address directly the relationships between different species of *Zosterophyllum* and closely allied taxa are needed to clarify the taxonomy of this iconic taxon and its evolutionary relationships.

## *Zosterophylls and lycophytes: taxonomy in the light of phylogenetic relationships*

# *Previous taxonomic schemes*. In Banks’ (1968) initial classification of early vascular plants, zosterophylls are placed in Subdivision Zosterophyllophytina of Division Tracheophyta (Table 3), which he separated from Subdivision Lycophytina, which included the lycopsids. Subsequently, based on the six genera included in the group at the time, Banks (1975) listed as defining characters of Zosterophyllophytina: leafless axes with isotomous to pseudomonopodial branching, laterally borne sporangia with distal line of dehiscence and central primary xylem strand with exarch maturation, consisting of tracheids with scalariform thickenings. Banks’ classification was maintained as additional species and genera were discovered. Gensel (1992), for instance, mentioned 18 zosterophyll genera, of which she included seven in a phylogenetic analysis. Addition of new genera broadened the morphological range of zosterophylls and showed that tracheid wall thickenings formed a specific pattern, documented initially in *Gosslingia* (Kenrick and Edwards, [1988](#_bookmark36)b) and termed G-type thickenings (Kenrick and Crane, 1997).

# In an influential study that included 23 species of unequivocal or putative zosterophyll affinity, Kenrick and Crane (1997) using a phylogenetic framework concluded that these did not form a coherent group. Thus, in their classification scheme, some of the taxa recognizable as zosterophylls using Banks’ criteria form a clade (termed Plesion Zosterophyllopsida or Zosterophyllaceae), while others are members of a more inclusive clade – Subdivision Lycophytina of Division Tracheophyta – but with poorly resolved relationships to each other and to Zosterophyllopsida. Overall, Kenrick and Crane’s analysis supports a paraphyletic status of a group that includes taxa traditionally recognized as zosterophylls, as well as the barinophytes and some taxa of putative or disputed zosterophyll affinity (*Nothia*, *Hsua*, *Huia*); on the other hand, the traditionally recognized zosterophylls are polyphyletic in their analysis (Table 3).

# For Zosterophyllopsida, Kenrick and Crane list as synapomorphies circinate apical development, two-rowed arrangement of the laterally attached sporangia and, putatively, elliptical cross-sectional shape of the xylem. Additionally, zosterophyllopsids share with all other members of Lycophytina the following synapomorphies: exarch xylem maturation, reniform, dorsiventral isovalvate sporangia borne laterally along axes, on short stalks, with distal dehiscence line featuring conspicuous cellular thickening.

# Gerrienne *et al.* (2016), in their phylogenetically framed taxonomic compilation, followed broadly Kenrick and Crane’s (1997) classification scheme. However, in their view, the Lycophytina, circumscribed by the same synapomorphies, is split strictly into two lineages (Table 3), Lycopsida and Zosterophyllopsida, the latter characterized by circinate growth and two-rowed arrangement of sporangia. Although not explicitly stated, this classification scheme implies that, contrary to Kenrick and Crane’s findings, all non-lycopsid lycophytes form a clade (Zosterophyllopsida) sister to the Lycopsida.

# In a phylogenetic treatment of the diverse Pragian Posongchong flora of China, Hao and Xue (2013) recovered the eight zosterophyll species (representing six genera) included in the analysis as a clade called Zosterophyllopsida, sister to *Adoketophyton* (Table 3), with which they share circinate apical development and tracheids with wall microperforations between scalariform thickenings (like those of G-type tracheids). The synapomorphies of Zosterophyllopsida include a rowed arrangement of sporangia along axes and elliptical or strap-shaped cross section of xylem that has exarch maturation and consists of tracheids with G-type wall thickenings. Hao and Xue’s results do not support a Lycophytina clade.

# In summary, the taxonomic schemes proposed previously for lycophytes explicitly or implicitly support one of two major patterns of relationships. In one of them, zosterophylls form or are part of a grade paraphyletic to the lycopsids. This pattern of relationships is implied in Banks’ (1968) evolutionary hypothesis, wherein lycopsids (termed Lycophytina) arise from among a zosterophyll (Zosterophyllophytina) plexus, and is supported by the results of Gensel (1992) and Nibbelink and Tomescu (2022) (Table 3). Similar but not entirely congruent with this pattern of relationships, Kenrick and Crane’s (1997) Lycophytina clade consists of a grade paraphyletic to the lycopsids (Lycopsida); within this grade, traditionally recognized zosterophylls are polyphyletic and only a subset of them form a clade (Zosterophyllopsida) (Table 3). In contrast to this pattern, the analyses of Hao and Xue (2013) do not recover the lycophytes as a clade; instead, they support a zosterophyll clade (Zosterophyllopsida) sister to a clade that includes the lycopsids (Lycopsida) and the euphyllophyte, each form separate clades (Table 3). A zosterophyll clade is also implied by Gerrienne *et al.*’s (2016) sister-group relationship between the Zosterophyllopsida and Lycopsida lineages within a more inclusive Lycophytina clade.

# *Evolutionary implications*

# *Tempo of evolution*. During the review process the question arose if and how Silurian occurrences of zosterophylls and lycopsids not included in our dataset might alter the evolutionary tempo trends outlined above. Such are the reports of *Baragwanathia* (Lang and Cookson, 1935) and *Parazosterophyllum* (McSweeney *et al.*, 2020) from Australia, and of *Zosterophyllum*, *Distichophytum* and putative *Bathurstia* from Arctic Canada (Kotyk *et al.*, 2002). The lycopsid *Baragwanathia* has been reported from Silurian Australian strata dated to the early Ludlow (Gorstian) (Garratt, 1978; Garratt and Rickards, 1984). While this would push the origin of the lycopsid lineage 6-7 million years deeper than projected by our results (Fig. 3), that would not be inconsistent with our prediction of an initial Silurian radiation that gave rise to all the major lycophyte clades during the early Ludlow or possibly earlier. However, the Gorstian age of that *Baragwanathia* occurrence has been questioned on solid grounds by Hueber (1992), who suggested that it may be in fact Pragian, which would bring *Baragwanathia* well within the stratigraphic range of the lycopsids already included in our analysis. Like *Baragwanathia* (if indeed Gorstian in age), the putative *Bathurstia* (reported as cf. *Bathurstia* sp.) and *Distichophytum* sp. of Kotyk *et al.* (2002), dated to the late Ludlow (Ludfordian), would be consistent with our prediction on the timing of the initial Silurian radiation. These occurrences would also suggest that the radiation that gave rise to most of the genus-level diversity within the major lycophyte clades may have pre-dated the Ludfordian. On the other hand, for *Parazosterophyllum*, dated to the Pridoli (McSweeney *et al.*, 2020) and *Zosterophyllum* sp. of Kotyk *et al.* (2002), not knowing where they fit phylogenetically makes is impossible to assess how their inclusion would affect our evolutionary tempo predictions. Nevertheless, their ages, no older than *Macivera*, suggest that they would not alter our predictions significantly. In summary, our evolutionary tempo predictions are conservative; the Silurian lycophyte occurrences not considered in our analysis would not affect our predictions of two major cladogenetic episodes and would only shift their minimum ages deeper in time by no more than 7-10 million years.

# *Mode of evolution*. Apical circination, known in many zosterophylls, probably evolved independently in several lineages of the LBLC: the *Discalis*+*Gosferia* group, in *Adoketophyton*, *Trichopherophyton* and *Bathurstia*, and in the Sawdoniaceae, where they are a synapomorphy of the clade (with a reversal in *Margophyton*) (Fig. 3, 5).

# Multicellular emergences on the surface of axes, typically roughly conical and sometimes referred to as enations, are supported as a synapomorphy of the Sawdoniaceae. Absence of such emergences from some Sawdoniaceae indicates character state reversal. In this context, we note that two such members that form a clade within the Sawdoniaceae – *Gosslingia* and *Thrinkophyton* – possess fine trichomes on their axes (Edwards, [1970](#_bookmark17); Kenrick and Edwards, [1988](#_bookmark43)a). The derived position of *Gosslingia*+*Thrinkophyton* among the Sawdoniaceae allows us to speculate that their trichomes could have evolved by reduction of enations.

# Our results also suggest a complex pattern of evolution of sporangium-bearing appendages among the lycophytes. On one hand, the phylogenetic placement of *Adoketophyton* far from other taxa with sporangium-bearing appendages suggests that the fan-shaped appendages associated with the sporangia in this genus evolved independently. On the other hand, the sister-group relationship between the *Bathurstia*+Barinophytace and the Sawdoniaceae+lycopsids clades, wherein *Bathurstia* and Sawdoniaceae bear enations, and Barinophytaceae and lycopsids possess sporangium-bearing appendages, allows us to speculate that the common ancestor of the *Bathurstia*+Barinophytace - Sawdoniaceae+lycopsids clade may have possessed enations that were ‘primed’ in some way to evolve into sporangium-bearing appendages, independently in the Barinophytace and the lycopsids. Such speculations are tempered by the observation that while in the derived lycopsids sporangia are borne adaxially on leaves, the plesiomorphic condition present in the drepanophycalean members of the lineage involves sporangia that are not necessarily borne on leaves but, instead, occur in the position of leaves (Schweitzer, 1980; Turner *et al.*, 2023).

# **References**

Arnold CA. 1939. Observations on fossil plants from the Devonian of eastern North America. IV. Plant remains from the Catskill Delta deposits of northern Pennsylvania and southern New York. *Contributions from the Museum of Paleontology, University of Michigan* 5: 271–314.

Banks HP. 1968. The early history of land plants. In: Drake ET, ed. *Evolution and environment*. New Haven: Yale University Press, 73–107.

Banks HP, Davis M. 1969. *Crenaticaulis*, a new genus of Devonian plants allied to *Zosterophyllum*, and its bearing on the classification of early land plants. *American Journal of* *Botany* 56: 436–449.

Banks HP. 1975. Reclassification of Psilophyta. *Taxon* 24: 401–413.

Berry CM, Edwards D. 1994. New data on the morphology and anatomy of the Devonian zosterophyll *Serrulacaulis* Hueber and Banks from Venezuela. *Review of Palaeobotany and* *Palynology* 81: 141–150.

Bonacorsi NK, Gensel PG, Hueber FM, Leslie AB. 2021. *Omniastrobus* gen. nov., an Emsian plant with implications for the evolution of heterospory in the Early Devonian. *International Journal of Plant Sciences* 182: 198–209.

Brauer DF. 1980. *Barinophyton citrulliforme* (Barinophytales incertae sedis, Barinophytaceae) from the Upper Devonian of Pennsylvania. *American Journal of Botany* 67: 1186–1206.

Brauer DF. 1981. Heterosporous barinophytacean plants from the Upper Devonian of North America and a discussion of the possible affinities of the Barinophytaceae. *Review of Palaeobotany and Palynology* 33: 347–362.

Crepet WL, Niklas KJ. 2019. The evolution of early vascular plant complexity. *International Journal of Plant Sciences* 180: 800–810.

Doran JB. 1980. A new species of *Psilophyton* from the Lower Devonian of northern New Brunswick, Canada. *Canadian Journal of Botany* 58: 2241–2262.

Edwards D. 1970. Further observations on the Lower Devonian plant, *Gosslingia breconensis* Heard. *Philosophical Transactions of the Royal Society of London B Biological Sciences* 258: 225–243.

Edwards D. 1975. Some observations on the fertile parts of *Zosterophyllum myretonianum* Penhallow from the lower Old Red Sandstone of Scotland. *Earth and Environmental Science* *Transactions of the Royal Society of Edinburgh* 69: 251–265.

Edwards D. 1976. The systematic position of *Hicklingia edwardii* Kidston and Lang. *New* *Phytologist* 76: 173–181.

Edwards D, Kenrick P. 1986. A new zosterophyll from the Lower Devonian of Wales. *Botanical* *Journal of the Linnean Society* 92: 269–283.

Edwards D, Li C-S. 2018a. Diversity in affinities of plants with lateral sporangia from the Lower Devonian of Sichuan Province, China. *Review of Palaeobotany and Palynology* 258: 98–111.

Edwards D, Li C-S. 2018b. Further insights into the Lower Devonian terrestrial vegetation of Sichuan Province, China. *Review of Palaeobotany and Palynology* 253: 37–48.

Edwards D, Kenrick P, Carluccio LM. 1989. A reconsideration of cf. *Psilophyton princeps* (Croft & Lang, 1942), a zosterophyll widespread in the lower Old Red Sandstone of South Wales. *Botanical Journal of the Linnean Society* 100: 293–318.

Edwards D, Geng B-Y, Li C-S. 2016. New plants from the Lower Devonian Pingyipu Group, Jiangyou County, Sichuan Province, China. *PLoS One* 11: e0163549.

Edwards DS. 1986. *Aglaophyton major*, a non-vascular land-plant from the Devonian Rhynie chert. *Botanical Journal of the Linnean Society* 93: 173–204.

Fairon M. 1967. L’*Asteroxylon elberfeldense* Kräusel et Weyland porte-t-il des axes terminaux du type *Hostimella hostimensis* Potonié et Bernard? *Annales de la Société géologique de* *Belgique* 10: 7–30.

Garratt MJ. 1978. New evidence for a Silurian (Ludlow) age for the earliest *Baragwanathia* flora. *Alcheringa* 2: 217–224.

Garratt MJ, Rickards RB. 1984. Graptolite biostratigraphy of early land plants from Victoria, Australia. *Proceedings of the Yorkshire Geological Society* 44: 377–384.

Gensel PG. 1982. *Oricilla*, a new genus referable to the zosterophyllophytes from the late Early Devonian of northern New Brunswick. *Review of Palaeobotany and Palynology* 37: 345–359.

Gensel PG. 1992. Phylogenetic relationships of the zosterophylls and lycopsids: evidence from morphology, paleoecology, and cladistic methods of inference. *Annals of the Missouri Botanical Garden* 79: 450–473.

Gensel PG, Andrews HN, Forbes WH. 1975. A new species of *Sawdonia* with notes on the origin of microphylls and lateral sporangia. *Botanical Gazette* 136: 50–62.

Gensel PG, Berry CM. 2016. Sporangial morphology of the Early Devonian zosterophyll *Sawdonia ornata* from the type locality (Gaspé). *International Journal of Plant Sciences* 177: 618–632.

Gerrienne P. 1991a. Les plantes emsiennes de Fooz-Wepion (bord nord du Synclinorium de Dinant, Belgique). I: *Anisophyton* cf. *gothanii* Remy, Schultka & Haas, 1986. *Bulletin de la* *Société belge de géologie* 100: 135–145.

Gerrienne P. 1991b. Les fossiles végétaux du Dévonien inférieur de Marchin (bord nord du Synclinorium de Dinant, Belgique). II. *Forgesia c*urvata gen. et sp. nov. *Comptes Rendus de l’Académie des Sciences, Paris* 313: 1213–1219.

Gerrienne P. 1996. Lower Devonian plant remains from Marchin (northern margin of Dinant Synclinorium, Belgium). IV. *Odonax borealis* gen. et sp. nov. *Review of Palaeobotany and* *Palynology* 93: 89–106.

Gerrienne P. 1999. *Gosferia*, a new name for *Forgesia* (fossil plants). *Taxon* 48: 61–61.

Gerrienne P, Servais T, Vecoli M. 2016. Plant evolution and terrestrialization during Palaeozoic times – the phylogenetic context. *Review of Palaeobotany and Palynology* 227: 4–18.

Göppert HR. 1852. Fossile Flora des Ubergangsgebirges. *Verhandlungen der Kaiserlichen Leopold-Carol Akademie, Naturwissenschaftliche Classe* 14: 1-299.

Hao S-G. 1989a. *Gumuia* – a new genus of the Lower Devonian from Yunnan. *Acta Botanica* *Sinica* 31: 954–961.

Hao S-G. 1989b. New zosterophyll from the Lower Devonian (Siegenian) of Yunnan, China. *Review of Palaeobotany and Palynology* 57: 155–171.

Hao S-G, Wang D, Beck CB. 2003. Observations on anatomy of *Adoketophyton subverticillatum* from the Posongchong Formation (Pragian, Lower Devonian) of Yunnan, China. *Review of Palaeobotany and Palynology* 127: 175–186.

Hao S-G, Xue J-Z. 2011. A new zosterophyll plant, *Ramoferis* gen. nov., from the Posongchong Formation of Lower Devonian (Pragian) of southeastern Yunnan, China. *Acta Geo**logica Sinica* 85: 765–776.

Hao S-G, Xue J-H. 2013. *The early Devonian Posongchong fiora of Yunnan: a contribution to an understanding of the evolution and early diversification of vascular plants*. Beijing: Science Press.

Hao S-G, Gensel PG, Wang D-M. 2001. *Polythecophyton* demissum, gen. et sp. nov., a new plant from the Lower Devonian (Pragian) of Yunnan, China and its phytogeographic significance. *Review of Palaeobotany and Palynology* 116: 55–71.

Hetherington AJ, Bridson SL, Jones AL, Hass H, Kerp H, Dolan L. 2021. An evidence-based 3D reconstruction of *Asteroxylon mackiei*, the most complex plant preserved from the Rhynie chert. *eLife* 10: e69447.

Hueber FM. 1971a. Early Devonian plants from Bathurst Island, District of Franklin. *Geological Survey of Canada Papers* 71-28: 1–11.

Hueber FM. 1971b. *Sawdonia ornata*: a new name for *Psilophyton princeps* var. ornatum. *Taxon* 20: 641–642.

Hueber FM. 1972. *Rebuchia ovata*, its vegetative morphology and classification with the Zosterophyllophytina. *Review of Palaeobotany and Palynology* 14: 113–127.

Hueber FM. 1992. Thoughts on the early lycopsids and zosterophylls. Annals of the Missouri Botanical Garden 79: 474–499.

Hueber FM, Banks HP. 1979. *Serrulacaulis furcatus* gen. sp. nov., a new zosterophyll from the lower Upper Devonian of New York State. *Review of Palaeobotany and Palynology* 28: 169–189.

Jensen DP, Gensel PG. 2013. *Forania plegiospinosa*, gen. et sp. nov.: a zosterophyll from the Early Devonian of New Brunswick, Canada, with a novel emergence type. *International* *Journal of Plant Sciences* 174: 687–701.

Kenrick P, Edwards D. 1988a. A new zosterophyll from a recently discovered exposure of the Lower Devonian Senni Beds in Dyfed, Wales. *Botanical Journal of the Linnean Society* 98: 97–115.

Kenrick P, Edwards D. 1988b. The anatomy of Lower Devonian *Gosslingia breconensis* Heard based on pyritized axes, with some comments on the permineralization process. *Botanical Journal of the Linnean Society* 97: 195–223.

Kenrick P, Crane PR. 1997. *Origin and early diversification of land plants*. Washington: Smithsonian Institution Press.

Kerp H, Wellman CH, Krings M, Kearney P, Hass H. 2013. Reproductive organs and in situ spores of *Asteroxylon mackiei* Kidston & Lang, the most complex plant from the Lower Devonian Rhynie chert. *International Journal of Plant Sciences* 174: 293–308.

Kidston R, Lang WH. 1920a. On Old Red Sandstone plants showing structure, from the Rhynie chert bed, Aberdeenshire. Part II. Additional notes on *Rhynia gwynne-vaughani*, Kidston and Lang; with descriptions of *Rhynia major*, n. sp., and *Hornea lignieri*, n. g., n. sp. *Earth and Environmental Science Transactions of the Royal Society of Edinburgh* 52: 603–627.

Kidston R, Lang WH. 1920b. On Old Red Sandstone plants showing structure, from the Rhynie chert bed, Aberdeenshire. Part III. *Asteroxylon mackiei*, Kidston and Lang. *Earth and Environmental Science Transactions of the Royal Society of Edinburgh* 52: 643–680.

Kidston R, Lang WH. 1924. Notes on fossil plants from the Old Red Sandstone of Scotland. I. *Hicklingia edwardi*, K. et L. *Earth and Environmental Science Transactions of* *the Royal Society of Edinburgh* 53: 405–407.

Kotyk ME. 1998. *Late Silurian and Early Devonian fossil plants of Bathurst Island, Arctic Canada*. (Doctoral dissertation). University of Saskatchewan, Canada.

Kotyk ME, Basinger JF. 2000. The Early Devonian (Pragian) zosterophyll *Bathurstia denticulata* Hueber. *Canadian Journal of Botany* 78: 193–207.

Kotyk ME, Basinger JF, Gensel PG, de Freitas TA. 2002. Morphologically complex plant macrofossils from the Late Silurian of Arctic Canada. *American Journal of Botany* 89: 1004–1013.

Lang WH, Cookson IC. 1935. On a flora, including vascular land plants, associated with *Monograptus*, in rocks of Silurian age, from Victoria, Australia. Philosophical Transactions of the Royal Society B: Biological Sciences 224: 421–449.

Lemoigne Y. 1970. Nouvelles diagnoses du genre *Rhynia* et de l’espèce *Rhynia gwynne-vaughanii*. *Bulletin de la Société botanique de France* 117: 307–320.

Li C-S, Edwards D. 1992. A new genus of early land plants with novel strobilar construction from the Lower Devonian Posongchong Formation, Yunnan Province, China. *Palaeontology* 35: 257–272.

Li C-S, Edwards D. 1995. A re-investigation of Halle’s *Drepanophycus spinaeformis* Göpp. from the Lower Devonian of Yunnan Province, southern China. *Botanical Journal of the* *Linnean Society* 118: 163–192.

Li C-S, Edwards D. 1996. *Demersatheca* Li et Edwards, gen. nov., a new genus of early land plants from the Lower Devonian, Yunnan Province, China. *Review of Palaeobotany and* *Palynology* 93: 77–88.

Lyon A, Edwards D. 1991. The first zosterophyll from the Lower Devonian Rhynie chert, Aberdeenshire. *Earth and Environmental Science Transactions of the Royal Society of Edin**burgh* 82: 324–332.

Matsunaga KKS, Cullen NP, Tomescu AMF. 2017. Vascularization of the *Selaginella* rhizophore: anatomical fingerprints of polar auxin transport with implications for the deep fossil record. *New Phytologist* 216: 419–428.

McSweeney F, Shimeta J, Buckeridge JSJ. 2020. Two new genera of early tracheophyta (Zosterophyllaceae) from the Upper Silurian–Lower Devonian of Victoria, Australia. *Alcheringa* 44: 379–396.

Morris J, Edwards D. 2014. An analysis of vegetational change in the Lower Devonian: new data from the Lochkovian of the Welsh Borderland, U.K. *Review of Palaeobotany and* *Palynology* 211: 28–54.

Mustafa H. 1978. Beiträge zur Devonflora III. *Argumenta Palaeobotanica* 5: 91–132.

Nibbelink M, Tomescu AMF. 2022. Exploring zosterophyll relationships within a more broadly sampled character space: a focus on anatomy. *International Journal of Plant Sciences* 183: 535–547.

Penhallow DP. 1892. Additional notes on Devonian plants from Scotland. *Canadian Record of Science* 5: 1–15.

Powell CL, Edwards D, Trewin NH. 1999. A new vascular plant from the Lower Devonian Windyfield chert, Rhynie, NE Scotland. *Earth and Environmental Science Transactions of the* *Royal Society of Edinburgh* 90: 331–349.

Rayner R. 1983. New observations on *Sawdonia ornata* from Scotland. *Earth and Environmental* *Science Transactions of the Royal Society of Edinburgh* 74: 79–93.

Remy W, Hass H. 1996. New information on gametophytes and sporophytes of *Aglaophyton major* and inferences about possible environmental adaptations. *Review of Palaeobotany* *and Palynology* 90: 175–193.

Remy W, Hass H, Schultka S. 1986a. *Anisophyton potoniei* nov. spec. aus den Kühlbacher Schichten (Emsian) vom Steinbruch Ufersmühle, Wiehltalsperre. *Argumenta Palaeobotan**ica* 7: 123–138.

Remy W, Schultka S, Hass H. 1986b. *Anisophyton gothani* nov. gen., nov. spec, und Hinweise zur Stratigraphie der Südlichen Wilbringhauser Scholle. *Argumenta Palaeobotanica* 7: 79–107.

Schweitzer H-J. 1980. Uber *Drepanophycus spinaeformis* Goeppert. *Bonner Palaobotanische Mitteilungen* 7: 1–29.

Schweitzer H-J. 1987. Introduction to the plant bearing beds and the flora of the Lower Devonian of the Rhineland. *Bonner Paläobotanische Mitteilungen* 13: 1–94.

Snigirevsky S, Tschibrikova E, Olli V. 2007. Fossil plants with spores in the sporangia from the Upper Devonian (Frasnian) deposits of northern Timan. *Paleontological Journal* 41: 461–468.

Turner HA, Humpage M, Kerp H, Hetherington AJ. 2023. Leaves and sporangia developed in rare non-Fibonacci spirals in early leafy plants. *Science* 380: 1188–1192.

Wang D-M, Hao S-G. 2001. A new species of vascular plants from the Xujiachong Formation (Lower Devonian) of Yunnan Province, China. *Review of Palaeobotany and Palynology* 114: 157–174.

Wang D-M, Hao S-G. 2002. *Guangnania cuneata* gen. et sp. nov. from the Lower Devonian of Yunnan Province, China. *Review of Palaeobotany and Palynology* 122: 13–27.

Wang Y, Bai J, Liu B-C, Wang Y, Xu H-H. 2022. New insights into the South China Lower Devonian flora based on fossils from Hezhang, Guizhou Province. *Philosophical* *Transactions of the Royal Society B* 377: 20210312.

Xu H-H, Berry CM, Wang Y. 2011. Morphological study on the Devonian zosterophyll *Serrulacaulis* Hueber and Banks: new materials and emendation. *Palaeoworld* 20: 322–331.

Xue J-H. 2009. Two zosterophyll plants from the Lower Devonian (Lochkovian) Xitun Formation of northeastern Yunnan, China. *Acta Geologica Sinica* 83: 504–512.

Zakharova T. 1981. On the systematic position of the species “*Psilophyton*” *goldschmidtii* from the Lower Devonian of Eurasia. *Paleontological Journal* 23: 109–118.

Zdebska D. 1982. A new zosterophyll from the Lower Devonian of Poland. *Palaeontology* 25: 247–263.

Zhu W-Q, Kenrick P. 1999. A *Zosterophyllum*-like plant from the Lower Devonian of Yunnan Province, China. *Review of Palaeobotany and Palynology* 105: 111–118.

Zhu X, Xue J-Z, Hao S-G, Wang D-M. 2011. A new species of *Adoketophyton* from the Lower Devonian (Pragian) Posongchong Formation of Yunnan, China. *Review of Palaeobotany and Palynology* 164: 238–246.
